# miR-193b-3p suppresses lung cancer cell migration and invasion through *PRNP* targeting

**DOI:** 10.1186/s12929-025-01121-1

**Published:** 2025-02-20

**Authors:** Hsiang-Ling Ho, Shin-Chih Lin, Chao-Wei Chiang, Ching Lin, Che-Wei Liu, Yi-Chen Yeh, Mei-Yu Chen, Teh-Ying Chou

**Affiliations:** 1 Department of Pathology and Laboratory Medicine, Taipei Veterans General Hospital, Taipei, 112201 Taiwan; 2 Department of Biotechnology and Laboratory Science in Medicine, National Yang Ming Chiao Tung University, Taipei, 112304 Taiwan; 3 Institute of Biochemistry and Molecular Biology, National Yang Ming Chiao Tung University, Beitou District, No.155, Section 2, Linong Street, Taipei, 112304 Taiwan; 4 Institute of Clinical Medicine, National Yang Ming Chiao Tung University, Taipei, 112304 Taiwan; 5 Faculty of Medicine, School of Medicine, National Yang Ming Chiao Tung University, Taipei, 112304 Taiwan; 6 Cancer Progression Research Center, National Yang Ming Chiao Tung University, Taipei, 112304 Taiwan; 7 Department of Pathology and Precision Medicine Research Center, Taipei Medical University Hospital, Taipei Medical University, Taipei, 110301 Taiwan; 8 Graduate Institute of Clinical Medicine, School of Medicine and Precision Health Center, Taipei Medical University, No. 250, Wuxing Street, Xinyi District, Taipei, 110301 Taiwan

**Keywords:** Lung cancer, Migration, Invasion, Metastasis, miR-193b-3p, Post-transcriptional regulation, *PRNP*, Cellular prion protein

## Abstract

**Background:**

Tumor metastasis is responsible for approximately 90% of mortality in lung cancer. Understanding the molecular mechanisms of lung cancer metastasis is crucial for developing new treatment strategies. Cellular prion protein (PrPc), encoded by *PRNP* gene, was previously found to enhance lung cancer invasiveness. However, research on the post-transcriptional regulation of *PRNP* remains limited.

**Methods:**

Dual-luciferase reporter assays identified miRNAs targeting the *PRNP* 3'-UTR, and RNA immunoprecipitation (RIP) confirmed the interaction between miR-193b-3p and *PRNP* mRNA. Promoter deletions and chromatin immunoprecipitation (ChIP) assays established c-Jun as a transcriptional repressor of miR-193b-3p. Functional validation of the c-Jun-miR-193b-3p-PrPc axis was conducted using transwell assays, LNA-in situ hybridization, RT-PCR, Western blot, and immunohistochemistry. Subcutaneous mouse xenograft models assessed the anti-tumor effects of miR-193b-3p in vivo.

**Results:**

We demonstrated that miR-193b-3p downregulates PrPc expression by directly targeting the 3'-UTR of *PRNP*. Overexpression of miR-193b-3p significantly suppressed *PRNP* expression at both mRNA and protein levels, and reduced lung cancer cell migration, invasion and proliferation, which was reversed by PrPc overexpression. Conversely, miR-193b-3p silencing enhanced *PRNP* expression as well as those oncogenic properties, which were mitigated by *PRNP* knockdown. Spearman correlation analysis revealed a significant negative association between miR-193b-3p and PrPc expression in lung cancer tissues (*p* = 0.017), and Kaplan–Meier survival analysis demonstrated that high PrPc (*p* = 0.039) or low miR-193b-3p (*p* = 0.027) expression correlated with poorer overall survival. Intra-tumoral injection of the miR-193b-3p mimic in mouse xenograft models significantly reduced tumor volume. In addition, c-Jun was identified as a transcriptional repressor of miR-193b-3p. Functional studies revealed that c-Jun knockdown inhibited lung cancer cell migration, invasion, and proliferation, effects that were reversed by either PrPc overexpression or miR-193b-3p inhibitor treatment. A significant association between PrPc and c-Jun expression in lung cancer tissues (*p* = 0.004) was observed. High expression of PrPc and/or c-Jun was found to be associated with poor overall survival of patients (*p* < 0.05).

**Conclusions:**

This study is the first to uncover a novel regulatory pathway where c-Jun acts as a transcriptional repressor of miR-193b-3p, leading to *PRNP* upregulation, which promotes lung cancer migration and invasion. This previously unrecognized c-Jun-miR-193b-3p-PrPc axis also provides valuable insights for the potential development of new therapeutic strategies against lung cancer metastasis through RNA-targeting technology.

**Supplementary Information:**

The online version contains supplementary material available at 10.1186/s12929-025-01121-1.

## Introduction

Lung cancer is a leading cause of global cancer-related mortality, with tumor metastasis contributing significantly to treatment failure and patient death [1]. Tumor metastasis involves a complex sequence of events, including primary tumor cell proliferation, infiltration into adjacent tissues, and migration through blood or lymphatic vessels to form secondary tumors in distant organs, thereby making it challenging to manage [2]. Therefore, unraveling the mechanisms driving lung cancer metastasis is crucial for the development of effective treatments.

The cellular prion protein (PrPc), encoded by the *PRNP* gene, is a cell surface glycosylphosphatidylinositol (GPI)-anchored protein consisting of 208 amino acids. It exhibits ubiquitous expression across all mammalian tissues, with a prominent presence in the central nervous system. Beyond its association with transmissible spongiform encephalopathy, emerging research indicates that PrPc also plays a role in tumor progression by promoting cancer cell proliferation, metastasis, and drug resistance across various cancer types, including gastric cancer, pancreatic cancer, colorectal cancer and etc. [3–7].

Our previous study demonstrated PrPc participated in enhancing lung cancer invasiveness and metastasis by facilitating lamellipodia formation and JNK signaling. This process involved the nuclear factor interleukin 3 regulated (NFIL3) protein acting as a transcriptional activator of PrPc. Given the pivotal role of PrPc in tumor progression, targeting its suppression emerges as a promising therapeutic strategy for cancer treatment, though research on the post-transcriptional regulation of *PRNP* remains limited.

MicroRNAs (miRNAs), a subset of endogenous, non-coding RNA molecules, play a crucial role in regulating a wide range of biological processes by modulating gene expression at the post-transcriptional level; dysregulation of miRNA has been implicated in numerous human diseases, including cancer [8]. MiRNAs are initially transcribed by RNA polymerase II (RNase II) into long primary transcripts known as pri-miRNAs. Subsequently, the pri-miRNAs are cleaved by the enzyme Drosha in conjunction with its regulatory protein DGCR8, leading to the generation of the precursor microRNAs (pre-miRNAs). Following nuclear export to the cytoplasm via RanGTP–exportin-5, the pre-miRNAs undergo further processing into mature miRNAs. Upon binding to their target mRNAs, miRNAs initiate translation inhibition, either through mRNA degradation or translational repression [9].

Remarkably, miRNA target recognition does not hinge on perfect complementarity, allowing a single miRNA to regulate multiple mRNAs, and conversely, a single mRNA can be targeted by multiple miRNAs [10]. This underscores the necessity for a finely tuned mechanism in post-transcriptional regulation, as aberrant miRNA expression can disrupt these regulatory mechanisms, resulting in significant phenotypic consequences. Dysregulation of miRNA expression can originate from various facets of regulatory mechanisms, encompassing transcriptional processes, epigenetic modifications, the influence of long noncoding RNAs, and RNA editing events [11].

In this study, we explored the post-transcriptional regulation of *PRNP* in lung cancer cells. Our research revealed that miR-193b-3p plays a crucial role as a regulator, suppressing *PRNP* expression and consequently inhibiting lung cancer cell proliferation, migration, and invasion. Additionally, we identified c-Jun as a transcriptional repressor responsible for downregulation of miR-193b-3p, leading to enhancing PrPc expression in lung cancer cells. Notably, high levels of PrPc and/or c-Jun are associated with poor overall survival in patients with lung adenocarcinomas. This study provides insights into the post-transcriptional regulatory mechanisms of PrPc and emphasizes miR-193b-3p as a potential therapeutic agent for mitigating lung cancer invasiveness and metastasis.

## Methods

### Cell lines and cell culture

Human lung adenocarcinoma cell lines CL1-1 and CL1-5 were kindly provided in 2002 by Professor Pan-Chyr Yang of the National Taiwan University; these cell lines were derivatives of the lung adenocarcinoma cell line CL1-0. CL1-1 and CL1-5 were subpopulations of CL1-0 cells with increasing invasiveness obtained by rounds of Matrigel-coated transwell selection [12] All lung cancer cell lines were maintained in RPMI (Thermo Fisher Scientific Inc., ref no. 31800022) supplemented with 10% fetal bovine serum (FBS). The 293 T human embryonic kidney cell line was maintained in DMEM (Thermo Fisher Scientific Inc., ref no. 12800017) supplemented with 10% FBS. All cells were cultured at 37 °C in a humidified atmosphere with 5% CO2. All culture media were supplemented with penicillin/streptomycin (Corning Inc., product no. 30–002-CI). Cells were not cultured for more than two months. Cells were seeded at 5 × 10^4^ cells/ml and treated with JNK inhibitor SP600125 (Cell Signaling, #8177) at the indicated times.

### Patient samples and tissue microarrays

Two cohorts of tissue microarrays (TMA) were used in this study. The tissue microarray was constructed as described previously [13]. Cohort 1 consisted of 398 patients diagnosed with stage I lung adenocarcinoma who underwent tumor resection at the Taipei Veterans General Hospital (VGH-Taipei) between 1995 and 2007. The analysis of Cohort 1 focused on studying the correlation between the expression levels of PrPc and miR-193b-3p. Cohort 2 comprised 199 patients with lung adenocarcinoma at various stages; these patients underwent tumor resection at VGH-Taipei between 2002 and 2006. Cohort 2 was specifically employed to conduct survival analysis. Overall survival (OS) was defined as the period between the surgical resection date and either the date of death or the last follow-up. Disease-free survival (DFS) was defined as the duration between diagnosis and the date of either recurrence or death. Patients whose cause of death was unrelated to their condition or was indeterminable were censored from the analysis. This study was approved by the Ethics Committee of VGH-Taipei, Taiwan.

### Immunohistochemistry

Paraffin-embedded 5-μm TMA sections were deparaffinized using xylene and subsequently rehydrated by immersion in decreasing concentrations of ethanol (twice in 100%, once in 90%, and once in 70%). Antigen retrieval was performed by heating sections in 0.1 M citrate, followed by treatment with 3% H2O2 to inhibit endogenous peroxidase activity. The sections were then incubated overnight at 4 °C with primary antibodies: anti-PrPc (1:100, Dako Cytomation) and anti-c-Jun (1:200, Cell Signaling Technology, cat# 9165). Following washing in phosphate-buffered saline (PBS), sections underwent incubation with peroxidase-labeled secondary antibodies for 1 h at room temperature. Subsequent steps included incubation with diaminobenzidine, washing, and counterstaining with hematoxylin. Evaluation of all staining was conducted by pathologists. For PrPc staining, intensity scores were categorized as follows: 0 (no staining), 1 (weakly positive), 2 (moderately positive), and 3 (strongly positive), while the percentage scores were designated as 0 (0%), 1 (≤ 10%), 2 (11–50%), and 3 (51–100%). The overall IHC score for each sample was calculated by multiplying the intensity score with the percentage score, resulting in a scale from 0 to 9. Similarly, c-Jun expression was assessed with scores of 0 (no staining), 1 (weakly positive), 2 (moderately positive), and 3 (strongly positive), and the resulting intensity score was directly multiplied by the observed percentage in the tissue.

### RNA oligonucleotides and locked nucleic acid–modified oligonucleotide probes

mirVana™ miRNA Inhibitor anti-miR-193b (Cat# 4464084) and anti-miRNA negative control (Cat# 4464076) oligonucleotides were purchased from Thermo Fisher Scientific Inc. The LNA miRNA scramble control probe (/5DigN/GTGTAACACGTCTATACGCCCA) (product no. 99004-01), positive control probe U6 snRNA (/5DigN/CACGAATTTGCGTGTCATCCTT) (product no. 99002-01) and hsa-miR-193b probe (/5DigN/AGCGGGACTTTGAGGGCCAGTT) (product no. 38611-01) were obtained from Exiqon Inc.

### LNA-in situ hybridization

LNA-in situ hybridization performed according to the manufacturer's instructions. Five-micrometer-thin sections of FFPE tissues slides were deparaffinized in xylene three times for 5 min each, followed by 5 min each in serial dilutions of ethanol (100%,100%, 95%, 95%) and three changes of DEPC–treated water. Slides were fixed with 4% paraformaldehyde in DEPC-PBS at room temperature for 20 min, rinsed twice with DEPC-PBS, and then treated with 400 μg/mL proteinase K at 37 °C for 15 min. Post proteinase K digestion, slides were washed twice with DEPC-PBS, three times with fresh DEPC-PBS, and subsequently subjected to hybridization in incubation chambers overnight at 37 °C. The hybridization mixture comprised 40 nM of scramble control, miR-193b, and 1 nM of U6 snRNA LNA probes (sourced from BioChain Inc., cat# K2191050). Following hybridization, slides were washed with 0.2 × SSC buffer containing 2% BSA at 4 °C for 10 min, followed by two washes with 1 × alkaline phosphatase (AP) buffer. An AP-conjugated anti-DIG antibody (BioChain Inc., cat# K2191050) in a 1:200 dilution was applied to the slides and left to incubate overnight at 4 °C. Subsequent to this step, the slides underwent two washes with 1 × DEPC-PBS for 5 min each and two washes with 1 × AP buffer for 5 min each. Finally, the slides were stained by incubating in an NBT/BCIP solution at room temperature for 6 h, followed by counterstaining with nuclear fast red and mounting.

### Plasmid construction

The 3’-UTR of the human *PRNP* gene and its deletion fragments were amplified from complementary DNA (cDNA) of CL1-5 cells using the specific primers listed in Supplementary Table S1. These PCR amplicons were then cloned into the XbaI site of the pGL3-control vector (Promega). To generate miRNA-expressing plasmids, precursor miRNAs were amplified from CL1-1 genomic DNA with a ~ 250 bp flanking sequence using the designated cloning primer sets mentioned in Supplementary Table S2. The PCR amplicon was then ligated into the pCMV-HA and pLVX-IRES-Neo vectors. To generate pGL3-control with a mutated miR-193b-3p binding site sequence on the 3’-UTR of *PRNP*, PCR mutagenesis was performed using two overlay primers (5’-ATCATGAGCCGTTGCTAATGCCACCGGTCAAAAAGTATAACAGC-3’ and 5’-TGGCATTAGCAACGGCTCATGATGAACTCAATCAAAGG −3’) with six nucleotide substitutions relative to the wild-type 3’-UTR of *PRNP.* For miR-193b-3p promoter and its subsequent deletion constructs, genomic DNA extracted from CL1-1 cells were subjected to PCR amplification using the specific primers listed in Supplementary Table S3. The PCR amplicons were then ligated into the pGL3-basic vector. Primers used for the construction of ETS1-, GR-, and c-Jun-expressing plasmids are detailed in Supplementary Table S2.

### Quantitative real-time PCR

Total RNA was isolated using the REzol^TM^ C&T reagent (Protech Technology Inc., cat# KP200CT). Complementary DNA (cDNA) was synthesized from 5 μg of total RNA utilizing the RevertAid First Strand cDNA Synthesis Kit (Thermo Fisher Scientific Inc., cat# K1622). Subsequently, equal amounts of cDNA were applied for the analysis of specific gene expression using the SensiFAST™ SYBR® Hi-ROX Kit (BIOLINE Inc., CSA-01068) in accordance with the manufacturer’s instructions. Primer sequences designed for assessing the expression of specific genes are provided in Supplementary Table S4.

### Luciferase assay

The 3’-UTR of *PRNP*, containing the putative miR-193b-3p binding site, and the promoter sequences of miR-193b-3p were amplified and cloned into the pGL3 Firefly Luciferase reporter vector. The pRL-TK *Renilla* Luciferase reporter vector was used as a normalization control. All reporter vectors were obtained from Promega Corporation (Madison, WI, USA). Mutations in the c-Jun binding sites within the reporter constructs were introduced by oligonucleotide-directed mutagenesis using the QuikChange® kit (Stratagene) and confirmed by sequencing. To evaluate the effects of miRNAs on the 3’-UTR of *PRNP*, reporter constructs with or without the 3’-UTR of *PRNP* were co-transfected with plasmids expressing miRNAs. To assess the repressive effect of c-Jun on the miR-193b-3p promoter, reporter constructs with either wild-type or mutant promoter sequences were co-transfected with plasmids expressing c-Jun. Transfections were performed using Lipofectamine LTX with Plus reagent (Thermo Fisher Scientific Inc., Cat. #15338100) according to the manufacturer’s instructions. Luciferase activity was measured 24 h post-transfection using the Luciferase Reporter System (Promega, Cat. #E1501), with *Renilla* luciferase (Promega, Cat. #E2820) as a control. Firefly luciferase activity was normalized to *Renilla* luciferase activity.

### Primary and mature MiRNA detection

The expression of mature miRNAs was assessed using TaqMan™ MicroRNA Assays (Assay ID: miR-22-3p = 000398; miR-193a-3p = 002250; miR-193b-3p = 002367; miR-216b-5p = 002326; miR-1290-3p = 002863; miR-653-5p = 002292) in accordance with the manufacturer’s instructions (Thermo Fisher Scientific Inc.). Briefly, reverse transcription reactions were performed using stem‒loop RT primers with the TaqMan™ MicroRNA Reverse Transcription Kit (Cat# 4,366,596) in a GeneAmp™ PCR System 9700 (Thermo Fisher Scientific Inc.). Real-time PCR was performed under the following conditions: 95 °C for 10 min, followed by 40 cycles at 95 °C for 15 s and 60 °C for 1 min by using TaqMan™ Universal Master Mix II, no UNG (Cat# 4,440,040). All samples were normalized to the internal control RNU6B. For primary miR-193b detection, equal amounts of cDNA were subjected to TaqMan™ Pri-miRNA Assay (Assay ID = Hs03303897_pri) following the manufacturer’s instructions, and all samples were normalized to the internal control, GAPDH (Assay ID = Hs99999905_m1).

### Western blotting

Total proteins were isolated using modified RIPA buffer (50 mM Tris; pH = 7.5, 150 mM NaCl, 0.1% SDS, 1% NP-40, 0.5% sodium deoxycholate) supplement with protease inhibitors (Merck Inc., cat# 04693132001) and/or phosphatase inhibitors (Sigma Inc., SI-P5726 and SI-P0044). Cells were lysed on ice for 30 min and the total lysate were subsequently centrifuged at 12,500 rpm for 10 min at 4 °C. The supernatant was collected, and subjected to protein quantification using Bradford Protein Assay Dye (Bio-Rad., cat# 5,000,006). Equal amounts of protein (50 µg) were subjected to 10% SDS-PAGE and transferred to polyvinylidene difluoride membranes (Pall Corporation). The membranes were then blocked with 5% non-fat milk in TBS at room temperature for 2 h and incubated at 4˚C overnight with primary antibodies, followed by incubation with horseradish peroxidase-conjugated secondary antibodies (GE Healthcare) for 2 h at room temperature. The primary antibodies used in this study were as follows: PrPc (Cayman, cat# 189720), α-tubulin (Sigma, product no. T9026), β-actin (Novus Biologicals, cat# NB600-501), GAPDH (Cell Signaling Technology, cat# 2118), phospho-JNK (Cell Signaling Technology, cat# 4668), total JNK (Cell Signaling Technology, cat# 9252), phospho-c-Jun (Cell Signaling Technology, cat# 9164) and c-Jun (Cell Signaling Technology, cat# 9165). Protein bands were detected by Western Chemiluminescent kit (HyCell biotechnology Inc.).

### Lentivirus transduction

Plasmids expressing shRNAs targeting *JUN* (TRCN0000039589, designated #1, and TRCN0000338165, designated #2) and *PRNP* (TRCN0000083492) were procured from the National RNAi Core Facility Platform of Academia Sinica, Taiwan. Virus packaging was carried out following previously established procedures [14]. Cells were cultured until reaching 60% confluence, and virus-containing media, supplemented with polybrene (8 μg/mL), were subsequently introduced into the culture medium. After 24 h of transduction, the virus-containing media were aspirated, and cells were subjected to selection with puromycin or hygromycin before being harvested for subsequent experiments.

### Chromatin immunoprecipitation (ChIP) assay

ChIP assays were conducted using the Magna ChIP™ A-Chromatin Immunoprecipitation Kit (Millipore, Cat. #17–610), following the manufacturer’s instructions. In brief, CL1-5 cells (5 × 10⁶) were crosslinked with 1% formaldehyde to preserve DNA–protein interactions, then quenched and washed with ice-cold PBS. The chromatin was sheared by sonication at 60 kHz for 30 cycles (30 s on, 30 s off) at 4 °C to generate fragments. Chromatin extracts were diluted tenfold with ChIP IP buffer and immunoprecipitated using an anti-c-Jun [E254] ChIP-grade antibody (Abcam, ab32137) or control IgG antibody (Cell Signaling Technology, #2729). Following immunoprecipitation, DNA fragments bound to the antibody were eluted, treated with proteinase K, and purified via phenol/chloroform extraction. Purified DNA was analyzed by quantitative PCR (qPCR) using primers targeting the predicted c-Jun binding site in the miR-193 promoter region, as listed in Supplementary Table S5.

### RNA immunoprecipitation (RIP) assay

RNA immunoprecipitation (RIP) assays were performed using the RIP Assay Kit (Cat. #17–700, Millipore) according to the manufacturer’s protocol. Briefly, CL1-1 cells (5 × 10⁶) were washed with ice-cold PBS and lysed in RIP lysis buffer containing a protease inhibitor cocktail and RNase inhibitor. The lysates were incubated overnight at 4 °C with magnetic beads conjugated to an AGO2-specific antibody (RN003M, MBL International) or control mouse IgG (Cat. #17–700, Millipore) with constant rotation. Following incubation and washing, the immunoprecipitated RNA was isolated by proteinase K treatment and phenol/chloroform extraction. The purified RNA was reverse transcribed, and the target RNAs (miR-193b-3p and *PRNP*) were assessed by qPCR analysis.

### In vitro migration and invasion assay

For the migration assay, Boyden chambers with filter inserts (8 μm pore size, Merck Millipore Ltd.) were positioned in 24-well plates. The lower well of each chamber was filled with medium containing 10% FBS, while approximately 2 × 10^4^ cells in culture medium with 1% FBS were seeded on top of the filter insert. Migration assays were conducted at 37 °C for 6 h. Subsequently, the chambers were fixed with 100% methanol for 20 min, air-dried, and stained with Giemsa (Sigma–Aldrich) for a minimum of 2 h. The invasion assay was performed as the migration assay, with the exception that the polycarbonate membranes were pre-coated with Matrigel (40 μg/100 μL serum-free medium; BD Bioscience, Bedford, MA) at 37 °C for 2 h to form the basement membrane. The invasion assay was conducted at 37 °C for 24 h. Following the assays, the membranes were imaged, and the migrated or invaded cells were quantified.

### Cell proliferation assay

Cells were seeded at a density of 1 × 10^4^ cells in 100 µL of culture medium per well into 96-well plates and then incubated for 72 h at 37 °C in a humidified atmosphere containing 5% CO2. Cell viability was assessed using the 3-(4,5-dimethylthiazol-2-yl)−2,5-diphenyltetrazolium bromide (MTT) assay, and the absorbance was measured at 570 nm using a Bio-Rad spectrophotometer (USA). Each experiment was conducted in quadruplicate and repeated at least three times.

### In vivo lung tumor model for miR-193b-3p treatment

The experimental procedure was performed as described previously with modifications [15]. Briefly, CL1-5 cells were mixed with Matrigel at a ratio of 1:1 and subcutaneously injected into 6- to 8-week-old NOD-SCID mice. When the tumor grew to 100–200 mm^3^, 20 μg of miRIDIAN microRNA Mimic Negative Control #1 (CN-001000–01–50) or miRIDIAN microRNA Human has-miR-193b-3p Mimic (C-300764–05–0050) (GE Healthcare Dharmacon, Inc.) were formulated with MaxSuppressor™ in vivo RNALancer II (BIOO Scientific, cat# 3410–01) following the manufacturer’s instructions, and formulated miRNA was intratumorally injected on days 1, 4 and 7. Tumor volume was evaluated every day by a caliper and was determined by the formula: V = 0.5 × a x b^2^, where a and b are the longest and the shortest diameter of the tumor, respectively.

### Statistical analysis

The data were analyzed using SPSS 22.0 (IBM Corp., Armonk, NY, USA) and Prism 7.0 (GraphPad, San Diego, CA, USA). Quantitative data are expressed as median (range) and were compared between groups using Student's t-test. Spearman rank correlation coefficient was used to evaluate the correlation between PrPc and miR-193b-3p, as well as between PrPc and c-Jun expression. Survival was analyzed using the Kaplan–Meier method and log-rank test. All statistical tests were two-sided, and p < 0.05 was considered statistically significant.

## Results

### MicroRNA identification in post-transcriptional regulation of *PRNP* in lung cancer

**Fig. 1:**
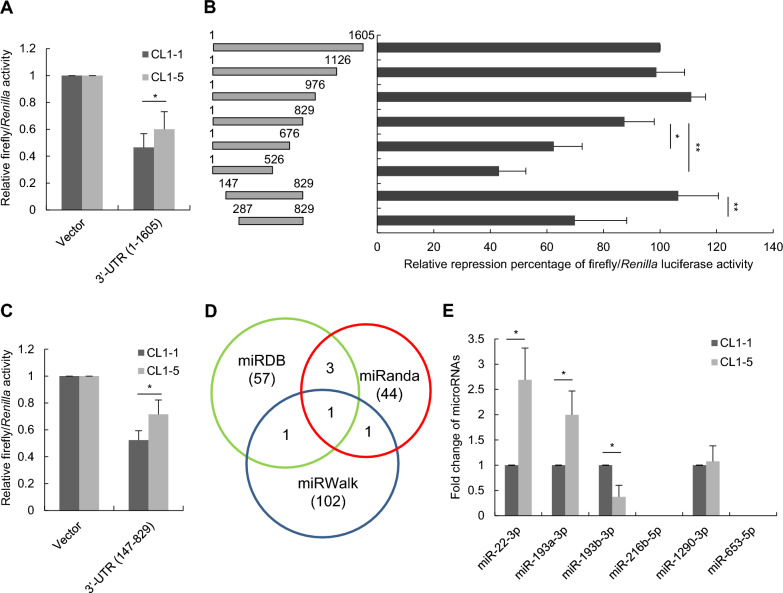
Identification of candidate miRNAs targeting 3’-UTR of *PRNP* in lung cancer. **A **The firefly reporter plasmid expressing the 3’-UTR of *PRNP* (1–1605) together with the *Renilla* reporter plasmid were transfected into CL1-1 and CL1-5 cells. The luciferase activity was determined 24 h post-transfection. **B **CL1-1 cells transfected with a series of 3’-UTR deletion reporter constructs of *PRNP*, were subjected to luciferase activity assays. The ratio of *Renilla*/firefly luciferase activities and the relative repression percentage were calculated. **C **Luciferase activity assays were conducted on CL1-1 and CL1-5 cells following transfection with the firefly reporter plasmid carrying 3’-UTR of *PRNP* (147–829), in combination with the *Renilla* reporter plasmid. **D **Pie chart illustrating the miRNAs predicted to target *PRNP* across three distinct databases, including miRDB, miRanda, and miRWalk. **E** The expression levels of miRNAs candidates were evaluated in CL1-1 and CL1-5 cells using stem-loop RT-qPCR assays. All quantitative data shown were means ± SD; * *p* < 0.05; ** p < 0.01, indicating statistical significance

To investigate the post-transcriptional regulation of *PRNP* in lung cancer cells, dual-luciferase reporter assays were conducted in CL1-1 and CL1-5 cells using plasmids containing the 3’-UTR of *PRNP* (1–1605) cloned into the firefly reporter vector. CL1-1 and CL1-5, lung adenocarcinoma cell lines with varying metastatic and invasive capabilities, have shown distinct levels of *PRNP* expression, with higher expression in the highly invasive CL1-5 cells [12]. Results revealed reduced luciferase activity in both CL1-1 and CL1-5 cells expressing the 3’-UTR of *PRNP* (1–1605) compared to vector controls (Fig. 1A). Interestingly, CL1-1 cells exhibited significantly lower luciferase activity than CL1-5 cells, suggesting predominant post-transcriptional regulation of *PRNP* in CL1-1 cells. To identify the critical region within the 3’-UTR of *PRNP*, CL1-1 cells transfected with a series of 3’-UTR deletion reporter constructs were subjected to luciferase activity assays. The 147–829 region within the 3’-UTR was identified as the shortest region with a suppressive effect similar to the full-length region (1–1605) (Fig. 1B and C), implying the presence of potential miRNA targeting sites in this region. The prediction tools of miRWalk, miRDB, and miRanda were then employed to screen potential *PRNP*-targeting miRNAs within this region, and the candidate miRNAs were selected if they were predicted by at least two different algorithms (Fig. 1D). Six putative miRNAs, including miR-193a-3p, miR-193b-3p, miR-1290-3p, miR-216b-5p, miR-653-5p, and miR-22-3p, were selected for further investigation (Supplementary Table S6). Endogenous expression levels of the six putative miRNAs were analyzed in both CL1-1 and CL1-5 cells (Fig. 1E). MiR-653-5p and miR-216b-5p were undetectable, miR-1290-3p exhibited no difference between CL1-1 and CL1-5, and miR-22-3p and miR-193a-3p displayed higher expression levels in CL1-5 than in CL1-1. Intriguingly, the expression level of miR-193b-3p was higher in CL1-1 than in CL1-5, suggesting that miR-193b-3p may serve as a potential negative regulator of *PRNP* in lung cancer cells.

### miR-193b-3p directly targeting *PRNP* 3’-UTR for expression suppression

**Fig. 2:**
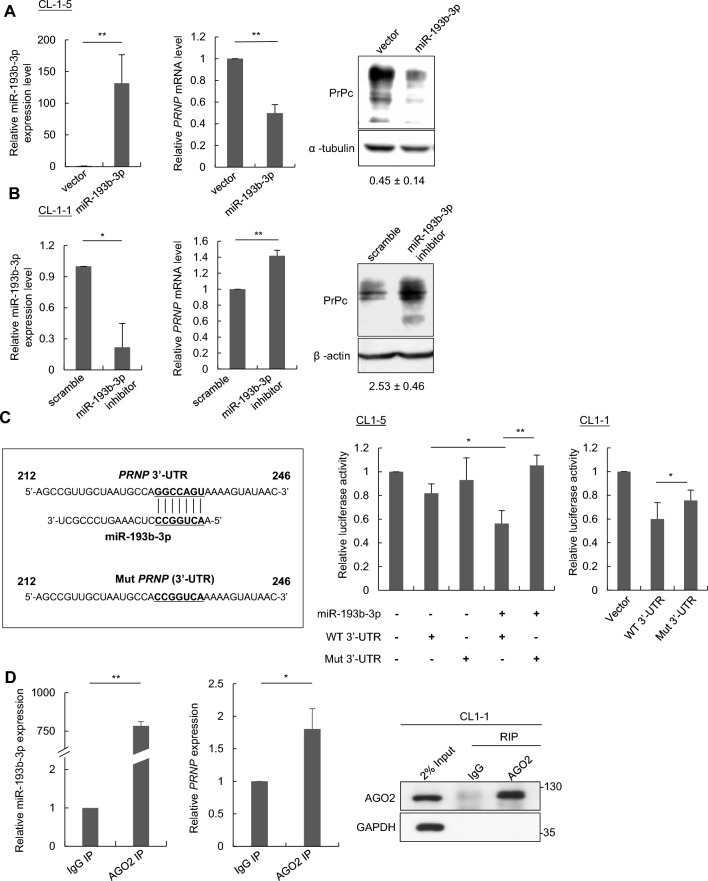

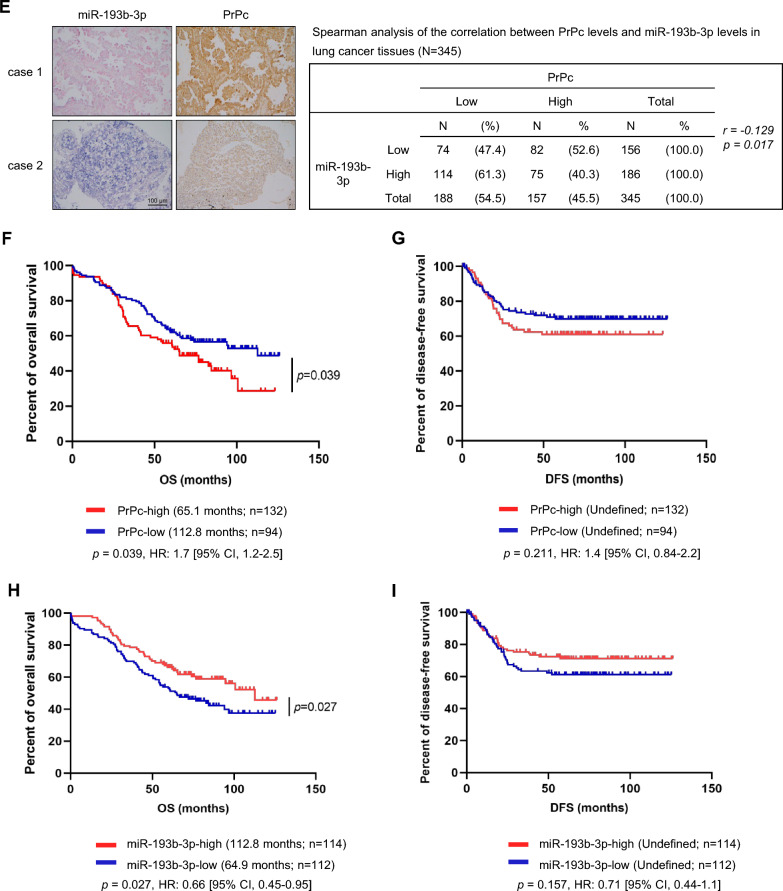
miR-193b-3p directly targets *PRNP* in lung cancer. **A** CL1-5 cells were transfected with a plasmid expressing miR-193b-3p or a vector control, and miR-193b-3p, *PRNP* mRNA, and protein levels were measured using stem-loop RT-qPCR, RT-qPCR, and Western blotting, respectively. **B** CL1-1 cells were transfected with anti-miR-193b-3p (miR inhibitors) or scramble control oligonucleotides, and miR-193b-3p, *PRNP* mRNA, and protein levels were measured using stem-loop RT-qPCR, RT-qPCR, and Western blotting, respectively. **C** Reporter plasmids expressing wild type (WT) or mutant (Mut) 3’-UTR of *PRNP* were co-transfected with or without the plasmid expressing miR-193b-3p into CL1-5 cells, and the luciferase activity was measured. Additionally, plasmids expressing WT or mutant 3’-UTR of *PRNP* reporter construct were transfected into CL1-1 cells to examine the endogenous effect of miR-193b-3p. **D** CL1-1 cells were subjected to RNA immunoprecipitation (RIP) analysis using an anti-AGO2 antibody, followed by RT-qPCR to assess the presence of miR-193b-3p and *PRNP* mRNA. Total lysates and immunoprecipitates were analyzed by Western blotting with anti-AGO2 and GAPDH antibodies. **E** The expression levels of miR-193b-3p and PrPc were examined in 345 stage I lung adenocarcinoma tissues using LNA-in situ hybridization and immunohistochemistry, respectively. Representative images were shown for case 1, indicating negative staining of miR-193b-3p and positive staining of PrPc, and case 2, showing positive staining of miR-193b-3p and negative staining of PrPc. Spearman correlation analysis revealed a significantly inverse correlation between miR-193b-3p and PrPc expression. **F** Overall survival for PrPc-high and PrPc-low. **G** Disease-free survival for PrPc-high and PrPc-low. **H** Overall survival for miR193b-3p-high and miR193b-3p-low. (**I**) Disease-free survival for miR193b-3p-high and miR193b-3p-low. All quantitative data shown were means ± SD; * *p* < 0.05; ** *p* < 0.01, indicating statistical significance

To delve deeper into the role of miR-193b-3p as a negative regulator of *PRNP* in lung cancer cells, miR-193b-3p expression levels were then manipulated in CL1-1 and CL1-5 cells to assess its impact on *PRNP* expression. In CL1-5 cells, miR-193b-3p overexpression reduced *PRNP* mRNA and protein expression levels (Fig. 2A). Conversely, silencing miR-193b-3p in CL1-1 cells with anti-miR-193b-3p oligonucleotides (miR-193b-3p inhibitors) increased *PRNP* expression at both mRNA and protein levels (Fig. 2B). These results showed a negative correlation between miR-193b-3p and *PRNP* expression in lung cancer cells. To examine whether the 3’-UTR of *PRNP* is a direct target of miR-193b-3p, two reporter constructs, one containing the wild-type 3’-UTR (WT 3’-UTR) of *PRNP* and the other with a mutated 3’-UTR sequence of *PRNP* (Mut 3’-UTR) were generated, and then co-transfected with or without miR-193b-3p into CL1-5 and CL1-1 cells for further analysis. Overexpression of miR-193b-3p effectively reduced the luciferase activity in cells expressing WT 3’-UTR but not Mut 3’-UTR of *PRNP* (Fig. 2C). CL1-1 cells expressing WT 3’-UTR but not Mut 3’-UTR also showed a reduced luciferase activity (Fig. 2C). To further confirm *PRNP* as a direct target of miR-193b-3p, we performed an RNA immunoprecipitation (RIP) assay in CL1-1 cells. The RNA-induced silencing complex (RISC) was pulled down using an AGO2-specific antibody, and the associated RNA was isolated for analysis of both miR-193b-3p and *PRNP* mRNA using reverse transcription quantitative PCR (RT-qPCR). As shown in Fig. 2D, both miR-193b-3p and *PRNP* mRNA were significantly enriched in the AGO2 immunoprecipitate compared to the IgG control, indicating that miR-193b-3p regulates *PRNP* expression by directly interacting with *PRNP* mRNA.

Moreover, we analyzed the expression levels of miR-193b-3p and PrPc in stage I lung adenocarcinoma samples using LNA-in situ hybridization (LNA-ISH) and immunohistochemistry (IHC), respectively. The staining results were initially scored on scales from 0 (no staining), 1 (low), 2 (medium), to 3 (high) (Supplementary Fig. S1). Scores 0 and 1 were categorized as low expression, while scores 2 and 3 indicated high expression. Spearman correlation analysis revealed a significant association between miR-193b-3p and PrPc expression in lung cancer tissues with a negative correlation observed (*p* = 0.017) (Fig. 2E). Moreover, Kaplan–Meier survival analysis indicated that high PrPc expression or low miR-193b-3p expression was significantly associated with poorer patient overall survival (OS) compared to cases with low PrPc expression (*p* = 0.039; Fig. 2F) or high miR-193b-3p expression (*p* = 0.027; Fig. 2H). However, we did not observe a significant association with disease-free survival (Fig. 2G and I).

Taken together, our findings reveal that miR-193b-3p downregulates PrPc expression by directly targeting the 3'-UTR of *PRNP*, potentially contributing to lung cancer progression.

### Role of miR-193b-3p in lung cancer cell migration and invasion through *PRNP*

**Fig. 3:**
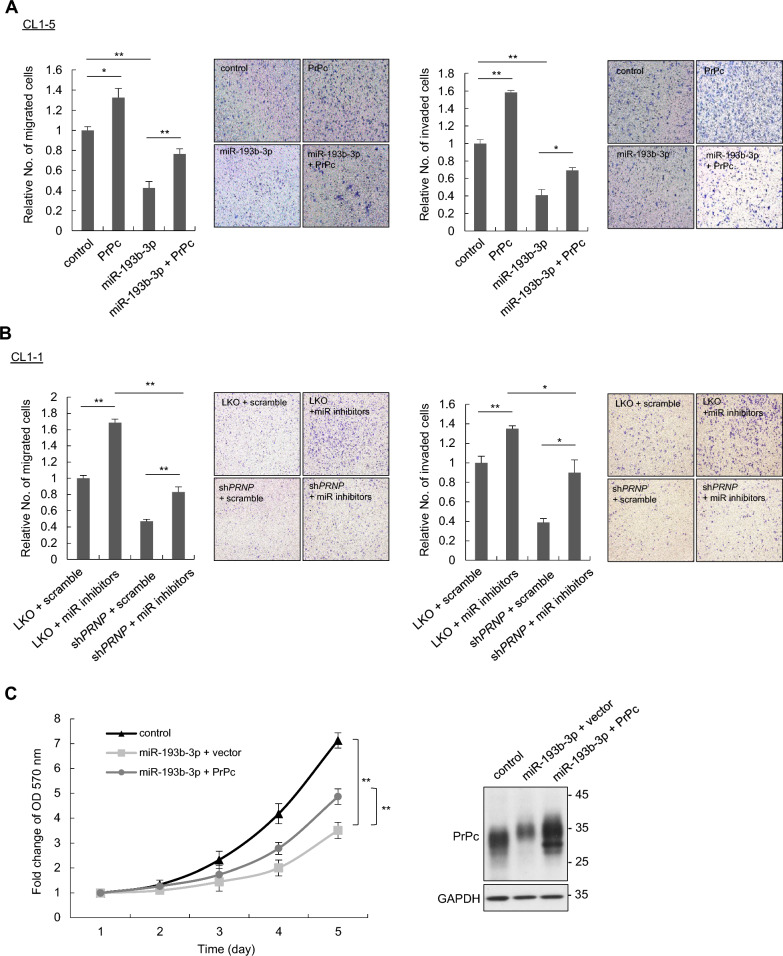

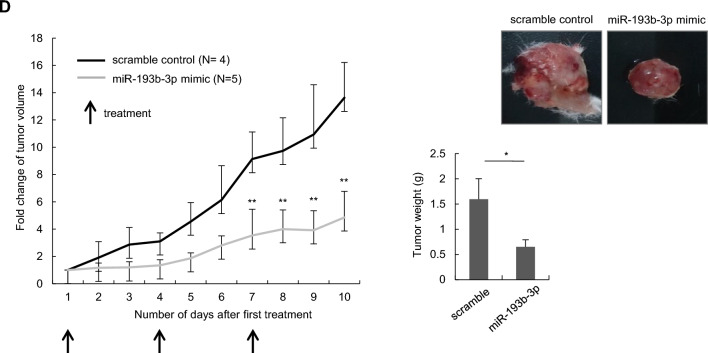
miR-193b-3p suppresses lung cancer migration, invasion and proliferation in vitro and in vivo. **A** CL1-5 cells, overexpressing control, PrPc and/or miR-193b-3p plasmids were subjected to transwell migration and invasion assays. **B** CL1-1 cells with LKO or *PRNP* silencing were transfected with anti-miR-193b-3p (miR inhibitors) or scramble control oligonucleotides, and then subjected to migration and invasion assays. **C** CL1-5 cells stably expressing either the vector control (pLVX) or miR-193b-3p were transfected with vector control (pCMV3-c-Myc) or PrPc and evaluated for cell proliferation using the MTT assay. All experiments were performed in triplicate. **D** CL1-5 cells were subcutaneously injected into NOD-SCID mice. After the tumor volumes reached 100–200 mm^3^, intra-tumoral administration of a miR-193b-3p mimic (N = 5) or the scramble control (N = 4) was carried out on day 1, 4, and 7. Tumor volume was evaluated daily. Representative images of tumors dissected from experimental mice were shown as indicated. All quantitative data shown were means ± SD; * p < 0.05; ** *p* < 0.01, indicating statistical significance

We subsequently investigated the impact of miR-193b-3p on PrPc-mediated cellular functions, including the proliferation, migration, and invasion capabilities of lung cancer cells. Overexpression of miR-193b-3p in CL1-5 cells resulted in reduced cell migration and invasion, while overexpression of PrPc reversed these effects (Fig. 3A). Similarly, miR-193b-3p inhibitor treatment in CL1-1 cells enhanced migration and invasion, which was mitigated by *PRNP* knockdown (Fig. 3B). Additionally, CL1-5 cells expressing miR-193b-3p showed reduced cell proliferation compared to those expressing the vector control (Fig. 3C and Supplementary Fig. S2), and this effect was reversed by PrPc overexpression (Fig. 3C). To further address the anti-tumor effect of miR-193b-3p in vivo, CL1-5 cells were subcutaneously injected into NOD-SCID mice, and when the tumor volumes reached 100–200 mm^3^, intra-tumoral administration of a miR-193b-3p mimic or a mimic negative control (NC) was performed on day 1, 4, and 7. As demonstrated in Fig. 3D, the mice treated with miR-193b-3p mimic exhibited a significant reduction of tumor volumes compared to those treated with NC. In summary, these results demonstrate that miR-193b-3p regulates cell migration and invasion through *PRNP* modulation, and that intra-tumor injection of the miR-193b-3p mimic effectively suppresses tumor growth in vivo in a xenograft model, indicating its potential as a therapeutic target for tumor progression.

### Transcriptional regulation of miR-193b-3p expression in lung cancer

**Fig. 4:**
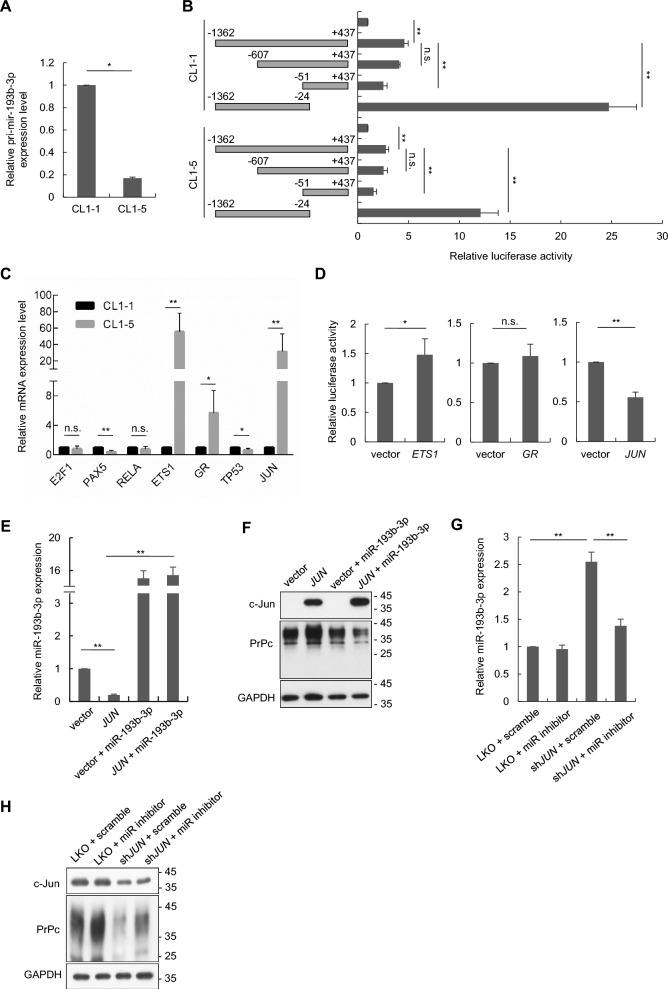
c-Jun is a transcriptional repressor of primary miR-193b in lung cancer. **A** The expression levels of pri-miR-193b-3p were assessed in CL1-1 and CL1-5 cells using RT-qPCR assays. **B** Dual-luciferase reporter assays were conducted for promoter deletion analysis. Firefly reporter plasmids expressing various deletion constructs of the miR-193b-3p promoter regions, along with the *Renilla* reporter plasmid, were transfected into CL1-1 and CL1-5 cells. Luciferase activity assays were performed 24 h post-transfection. **C** RT-qPCR assays were employed to analyze the expression levels of predicted transcription factors of miR-193b-3p in CL1-1 and CL1-5 cells. **D** Plasmids expressing *ETS1*, *GR*, or *JUN* were transfected into CL1-1 cells along with a luciferase reporter system of the miR-193b-3p promoter region (−1362 to + 437). Luciferase activity was measured to evaluate the impact of each transcription factor on miR-193-3p promoter activation. **E**–**F** CL1-1 cells overexpressing vector control, *JUN*, and/or miR-193b-3p were analyzed for miR-193b-3p expression by RT-qPCR (**E**) and PrPc expression by Western blotting (**F**). (**G**-**H**) CL1-5 cells with LKO or *JUN* knockdown were transfected with anti-miR-193b-3p (miR inhibitors) or scramble control oligonucleotides, followed by assessment of miR-193b-3p expression by RT-qPCR (**G**) and PrPc expression by Western blotting (**H**). All quantitative data shown were means ± SD; * p < 0.05; ** *p* < 0.01, indicating statistical significance

Considering that the expression level of miR-193b-3p is higher in CL1-1 cells than in CL1-5 cells (Fig. 1E), it is speculated that there may exist transcriptional mechanisms regulating miR-193b-3p expression. It has been known that miRNAs are initially transcribed to long primary miRNAs (pri-miRNAs) which undergo processing to become mature miRNAs, we therefore examined the expression level of primary miR-193b (pri-miR-193b) in CL1-1 and CL1-5 cells using RT-qPCR. The results showed that pri-miR-193b-3p expression levels were relatively lower in CL1-5 than CL1-1 cells (Fig. 4A). By performing promoter deletion analysis using a dual-luciferase reporter system, we found deletion of miR-193b-3p promoter region from −51 to + 437 significantly increased the luciferase activity in both CL1-1 and CL1-5 cells, suggesting the presence of a potential repressive element within this region (Fig. 4B). Bioinformatics approaches, utilizing prediction tools such as TFBIND, PROMO, and CONSITE, to identify putative transcription factor binding sites within the −51 to + 437 region, were conducted. The transcription factors predicted by these prediction tools were further compared with the differentially expressed genes analyzed from the cDNA microarray results of CL1-1 and CL1-5 cells, as well as with the UCSC ChIP database. Through these strategies, seven potential candidates, including *E2F1*, *PAX5*, *RELA*, *ETS1*, *GR*, *TP53*, and *JUN* were identified for subsequent analysis.

The mRNA expression levels of *ETS1*, *GR*, and *JUN* were found to upregulated in CL1-5 than in CL1-1 cells (Fig. 4C), implying these three transcription factors may participate in repressing miR-193b-3p expression. To further investigate this point, plasmids overexpressing *ETS1*, *GR*, or *JUN* were co-transfected with a luciferase reporter containing the miR-193b-3p promoter into CL1-1 cells. As shown in Fig. 4D, overexpression of c-Jun significantly reduced the promoter activity of miR-193b-3p, suggesting that c-Jun may act as a negative regulator of miR-193b-3p expression. To further investigate whether c-Jun regulates PrPc expression through modulation of miR-193b-3p, overexpression of *JUN* in CL1-1 cells significantly decreased the expression of pri-miR-193b-3p (Supplementary Fig. S3A) and miR-193b-3p (Fig. 4E), while increasing PrPc expression (Fig. 4F). Overexpression of miR-193b-3p under a CMV promoter reversed the increase in PrPc expression induced by *JUN* overexpression (Fig. 4F). Conversely, knockdown of *JUN* in CL1-5 cells resulted in an increase in pri-miR-193b-3p (Supplementary Fig. S3B) and miR-193b-3p (Fig. 4G) expression, along with a decrease in PrPc expression (Fig. 4H), which was partially reversed by treatment with miR-193b-3p inhibitors. In the control group (LKO), no obvious difference was observed between the scramble and miR-193b-3p inhibitor treatments, possibly due to that low miR-193b-3p expression in CL1-5 cells limited the detection of changes in miR-193b-3p levels following inhibitor treatment (Fig. 4G); nonetheless, an increase in PrPc expression was still observed (Fig. 4H).

Taken together, these findings demonstrated that c-Jun functions as a negative transcriptional regulator of miR-193b-3p, resulting in the upregulation of PrPc expression in lung cancer.

### c-Jun as a repressor of miR-193b-3p in PrPc-driven migration and invasion

**Fig. 5:**
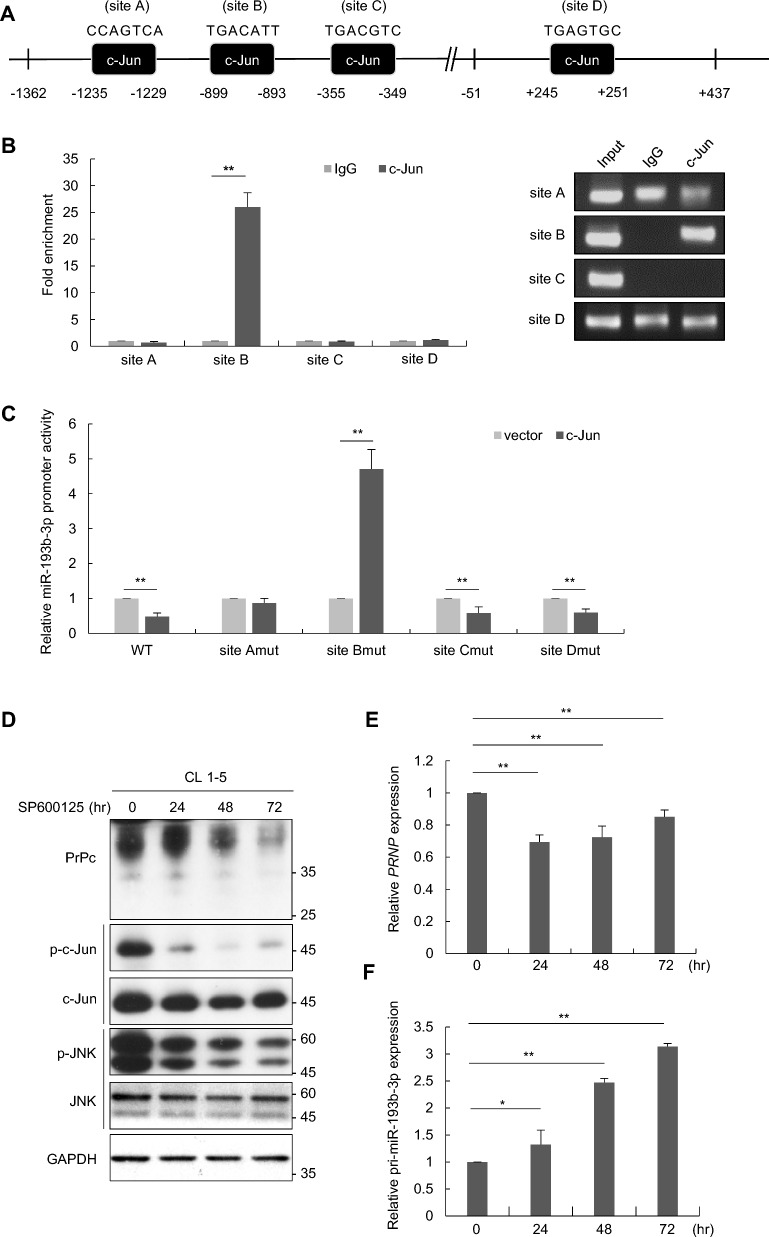

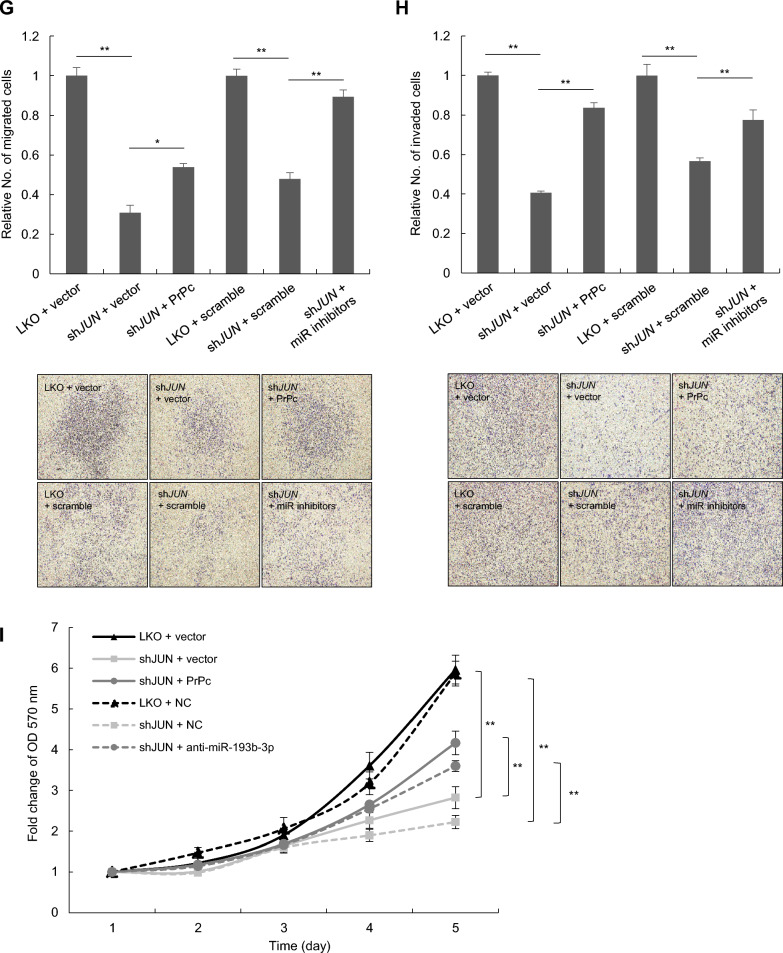
c-Jun binding to the miR-193b-3p promoter regulates miR-193b-3p and PrPc expression, influencing lung cancer cell migration and invasion. **A** Schematic representation of the miR-193b-3p promoter region, showing four predicted c-Jun binding sites: site A (−1235 bp to −1229 bp), site B (−899 bp to −893 bp), site C (−355 bp to −349 bp), and site D (+ 245 bp to + 251 bp). **B** Chromatin immunoprecipitation (ChIP) assay was performed using an anti-c-Jun antibody or a IgG control. The bound genomic DNA was extracted and analyzed by qPCR with primers specific to sites A, B, C, and D. **C** The luciferase constructs with site-specific mutations were transfected into CL1-1 cells, followed by luciferase assays with c-Jun or vector control overexpression. **D**–**F** CL1-5 cells were treated with the JNK inhibitor SP600125 for 0, 24, 48, and 72 h. At each time point, total cell lysates were collected for Western blot analysis using specific antibodies (**D**), and RNA was extracted for RT-qPCR to measure the expression levels of *PRNP* (**E**) and pri-miR-193b-3p (**F**). **G**-**H** CL1-5 cells with LKO or *JUN* knockdown were overexpressed with vector control, PrPc, or transfected with anti-miR-193b-3p (miR inhibitors) or scramble control oligonucleotides, and then subjected to transwell migration (**G**) and invasion (**H**) assays to assess cell motility. **I** CL1-5 cells with LKO or *JUN* knockdown were overexpressed with vector control, PrPc, or transfected with anti-miR-193b-3p (miR inhibitors) or scramble control oligonucleotides and subjected to the MTT assay to evaluate cell proliferation. All experiments were performed in triplicate. All quantitative data shown were means ± SD; * *p* < 0.05; ** *p* < 0.01, indicating statistical significance

To investigate whether c-Jun represses miR-193b-3p transcription by directly binding to its putative promoter region, a chromatin immunoprecipitation (ChIP) assay coupled with qPCR was performed. The four potential c-Jun binding sites within the miR-193b-3p promoter region were selected for analysis, including site A (−1235 bp to −1229 bp), site B (−899 bp to −893 bp), site C (−355 bp to −349 bp), and site D (+ 245 bp to + 251 bp) (Fig. 5A). The results revealed that, compared to the IgG control, c-Jun is predominantly bound to the site B region (Fig. 5B). To further explore the importance of these binding sites in repressing miR-193b-3p expression, luciferase constructs with site-specific mutations were transfected into CL1-1 cells, followed by luciferase assays upon c-Jun overexpression. As shown in Fig. 5C, c-Jun overexpression significantly reduced luciferase activity in the wild-type (WT), site C, and site D mutant constructs, whereas mutation at site A partially alleviated suppression and mutation at site B led to a significant increase of luciferase activity. Furthermore, inhibition of c-Jun activation with the JNK inhibitor SP600125 led to downregulation of both protein and mRNA levels of *PRNP* (Fig. 5D and E) and upregulation of pri-miR-193b-3p expression (Fig. 5F). These findings demonstrated that c-Jun directly binds to the miR-193b-3p promoter in lung cancer cells, with the binding site at position −899 bp to −893 bp being essential for the transcriptional repression of miR-193b-3p expression.

To further investigate whether c-Jun participates in regulating lung cancer migration and invasion through miR-193b-3p and PrPc, we conducted transwell migration and invasion assays, manipulating the expression of c-Jun, PrPc, and miR-193b-3p in CL1-5 cells. Our results indicate that silencing of c-Jun expression significantly inhibits cell migration and invasion compared to the control; overexpression of PrPc or treatment with miR-193b-3p inhibitors effectively reverses this inhibitory effect (Fig. 5G and H). Furthermore, *JUN* knockdown in CL1-5 cells resulted in diminished proliferation, which was restored by either PrPc overexpression or miR-193b-3p inhibitor treatment (Fig. 5I). In summary, these results indicated the involvement of the c-Jun-miR-193b-3p-PrPc axis in regulating lung cancer metastasis.

### Association of elevated c-Jun and/or PrPc with poor patient prognosis in lung cancer

**Fig. 6:**
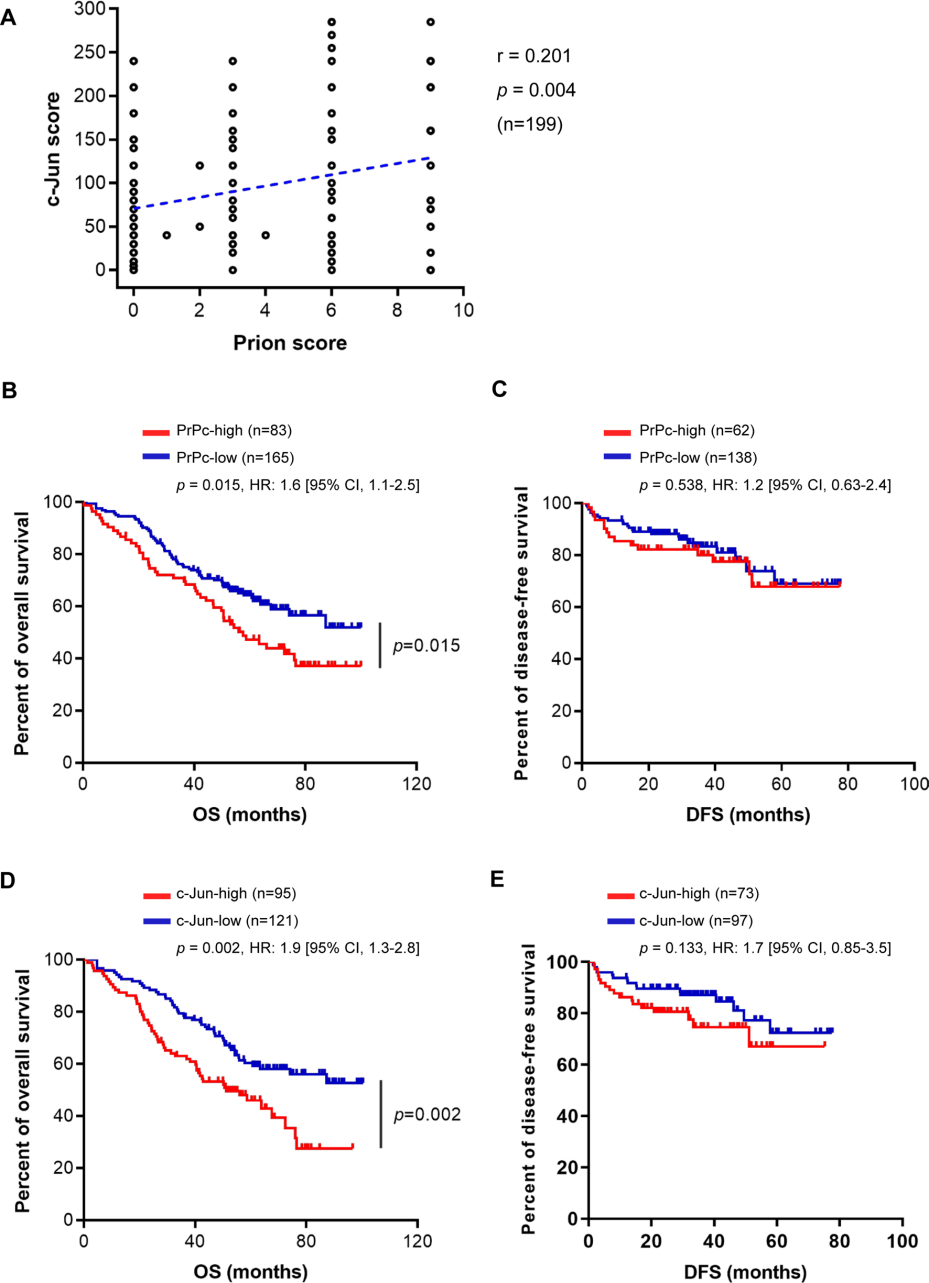

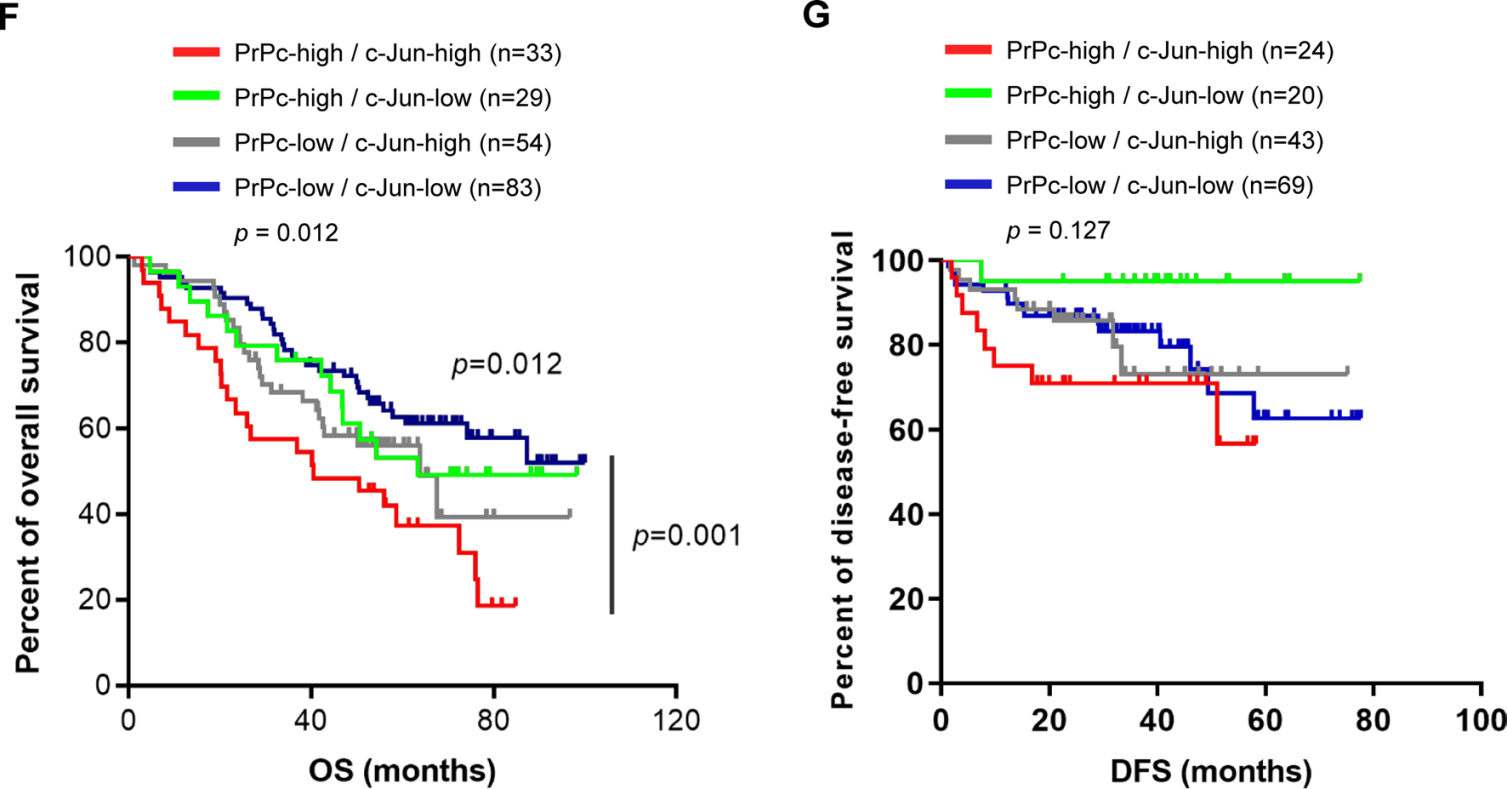
High expression of c-Jun or PrPc correlates with poor prognosis in patients with lung adenocarcinomas. A panel of 199 lung adenocarcinoma tissues was subjected to perform immunohistochemistry for c-Jun and PrPc. **A** Spearman correlation analysis was conducted to assess the expression relationship between c-Jun and PrPc. Kaplan–Meier survival analyses were performed to evaluate overall survival and disease-free survival for PrPc-high and PrPc-low (**B**, **C**), for c-Jun-high and c-Jun-low (**D**, **E**), and for PrPc-high/c-Jun-high, PrPc-high/c-Jun-low, PrPc-low/c-Jun-high, PrPc-low/c-Jun-low (**F**, **G**). *p* < 0.05, indicating statistical significance

Given the findings of c-Jun as a transcriptional repressor of miR-193b-3p involved in suppressing PrPc expression as well as its downstream functions in lung cancer cells, we then examined the in vivo correlation between c-Jun and PrPc by IHC staining of tumor tissues obtained from patients with lung adenocarcinomas. Spearman analysis revealed a positive correlation between c-Jun and PrPc expression in lung adenocarcinomas (*r* = 0.20; *p* = 0.004; Fig. 6A). Kaplan–Meier survival analysis indicated that patients with high levels of either c-Jun (c-Jun-high) or PrPc (PrPc-high) had significantly poorer overall survival (OS) compared to those with low levels of c-Jun (c-Jun-low) or PrPc (PrPc-low) (Fig. 6B for PrPc, *p* = 0.015; Fig. 6D for c-Jun, *p* = 0.002). Upon further characterization of IHC scores into four subgroups including PrPc-high/c-Jun-high, PrPc-high/c-Jun-low, PrPc-low/c-Jun-high and PrPc-low/c-Jun-low, patients with PrPc-high/c-Jun-high displayed the most adverse OS outcomes compared to other subgroups (*p* = 0.012) (Fig. 6F). However, no statistically significant difference was observed in disease-free survival (DFS) among these groups (Fig. 6C, E and G). Together, these data suggested high expression of c-Jun and/or PrPc associated with poor prognosis, implying the potential significance of c-Jun and PrPc as prognostic markers in lung adenocarcinomas.

## Discussion

The present study demonstrated, for the first time, a post-transcriptional regulatory mechanism of cellular prion protein expression, exerting a negative regulatory function in lung cancer migration and invasion. We provided evidence showing that 1) miR-193b-3p downregulates *PRNP* expression by directly targeting 3’-UTR of *PRNP*, leading to inhibit cell migration and invasion in lung cancer cells; 2) Injection of miR-193b-3p mimic in mouse xenografts significantly suppresses tumor growth in vivo, highlighting a potential anti-tumor effect of miR-193b-3p in lung cancer; 3) c-Jun serves as a transcriptional repressor of pri-miR-193b-3p, leading to upregulating *PRNP* expression; 4) high expression of PrPc and/or c-Jun are associated with poor patient’s overall survival, implying the prognostic potential of PrPc and c-Jun in lung cancer. In summary, these findings highlight miR-193b-3p may be a promising therapeutic target for mitigating lung cancer invasion and metastasis.

Activating the invasion and metastasis processes are known as one of cancer hallmarks, which enables cancer cells to invade local tissue and spread to distant organs, making surgical removals and therapeutic interventions more challenging or obstructive in cancer patients. Approximately 90% of cancer-related deaths are linked to metastases, therefore, exploring the mechanisms and molecular components involved in the mobility of cancer cells may offer new possibilities for developing novel strategies in cancer treatment [16].

The involvement of PrPc in cancer invasion and metastasis has been addressed in several cancer types [17, 18]. In colorectal cancers, the expression of PrPc was observed to be particularly concentrated at the invasive front of the tumor, where epithelial-mesenchymal transition (EMT) is prominent [6]. PrPc induced EMT in colorectal carcinoma cells, affecting the expression levels of E-cadherin, N-cadherin, and the translocation of β-catenin [6]. Additionally, PrPc was implicated in the metastatic potential of these cells by activating the Fyn-SP1-SATB1 pathway [6]. Overexpression of PrPc in colon cancer cell lines was associated with enhanced adhesion to fibronectin and collagen in the extracellular matrix, promoting both the growth and motility of cancer cells [19]. In breast cancer cell lines, elevated PrPc expression led to increased expression of *MMP9* at both mRNA and protein levels through the activation of NF-κB and ERK pathways, contributing to the heightened motility of breast cancer cells [20]. In terms of gastric cancer, PrPc was highly expressed in metastatic gastric cancer, contributing to cancer invasion via ERK1/2 pathway and is associated with increased expression of *MMP11* at both mRNA and protein levels [5]. In addition, PrPc was also found to associated to gastric cancer spreading to the liver and lymph nodes [5].

Regarding lung cancer, we previously demonstrated PrPc plays a critical role in lung cancer invasive and metastasis through regulating lamellipodium formation and cell mobility via JNK signaling pathway [14]. By analyzing lung cancer cells with varying invasive capabilities, specifically CL1-1 for low invasiveness and CL1-5 for high invasiveness, we observed a positive correlation between the expression level of PrPc and the invasiveness of tumor cells. However, the mechanisms responsible for differential expression of PrPc in lung cancer cells remain largely unknown.

MicroRNAs (miRNAs) are a class of small endogenous non-coding RNAs that exhibit a high degree of conservation across different species. They exert their regulatory influence by binding to the 3' -UTR of target genes, leading to the downregulation of gene expression. As a result, miRNAs play a pivotal role in modulating various cellular activities and processes [21]. Emerging evidence have shown the involvement of miRNAs in cancer metastasis, with their ability to either facilitate or impede metastasis through various mechanisms. These mechanisms include the regulation of oncogenes, tumor suppressor genes, metastasis-related genes and modulation of epithelial-mesenchymal transition as well as the tumor microenvironment processes [22, 23].

In the present study, by constructing a series of luciferase reporter plasmids containing various regions of 3’-UTR of *PRNP*, analyzing the luciferase activities in both CL1-1 (low invasiveness) and CL1-5 (highly invasiveness) lung cancer cells, we identified four potential *PRNP* targeting miRNAs that differentially expressed in CL1-1 and CL1-5 (Fig. 1E). Among these four miRNAs, the expression of miRNA-193b-3p showed a negative correlation with PrPc levels in CL1-1 and CL1-5 cells as well as in clinical lung cancer tissues (Fig. 2E). Mutation of the miR-193b-3p binding site within the 3’-UTR of *PRNP* was sufficient to abolish the inhibitory effect induced by miR-193b-3p (Fig. 2C). Additionally, RIP assays using an AGO2 antibody revealed that miR-193b-3p and *PRNP* mRNA co-enriched within the RISC complex (Fig. 2D). Our findings provide evidence that *PRNP* is a direct target of miR-193b-3p in lung cancer. Interestingly, a previous study using a genome-wide screening method to search for putative miRNAs that target *PRNP* in human neuroectodermal cell lines also found miR-193b-3p as one of the possible miRNAs that might negatively regulate the 3’-UTR of *PRNP* [24]. However, this finding was not further explored in the initial screening.

Numerous studies have elucidated the tumorigenic functions of miR-193b in various types of cancer, including breast cancer, gastric cancer, non-small cell lung cancer (NSCLC), and colorectal cancer [25]. However, the specific involvement of miR-193b-3p in regulating *PRNP* expression, particularly in lung cancer, has not been fully explored. MiR-193b was demonstrated to negatively regulate tumor migration, invasion and metastasis through targeting urokinase type plasminogen activator (*PLAU*), dimethylaminohydrolase 1 (*DDAH1*), cyclin D1 (*CCND1*) and *ETS1* in several cancer cell lines [26–30]. In breast cancer and pancreatic cancer cells, overexpression of miR-193b significantly reduced uPA protein expression and inhibited cell migration as well as invasion [31, 32]. Moreover, Hulin et al. have discovered an inverse correlation between miR-193b and *DDAH1* expression. Knockdown of *DDAH1* suppressed cell migration, implying miR-193b regulates cell migration through downregulating *DDAH1* expression (33). In HCC cell lines, by regulating the expression of *CCND1* and *ETS1*, miR-193b suppressed cell proliferation and colony formation, and inhibited the invasion and migration of cells [29].

Our study presents the first evidence of miR-193b-3p negatively regulating *PRNP* at the post-transcriptional level, thereby influencing tumor migration and invasion in lung cancer. By using two lung adenocarcinoma cell lines, CL1-1 and CL1-5, we observed that ectopic expression of miR-193b-3p in *PRNP*-high-expressing CL1-5 cells resulted in downregulation of cell migration and invasion, an effect that was partially reversed by re-overexpression of *PRNP* (Fig. 3A); depletion of miR-193b-3p in *PRNP*-low-expressing CL1-1 cells led to upregulation of cell migration and invasion, which was significantly reduced by knockdown of *PRNP* (Fig. 3B). These results demonstrate that miR-193b-3p, by modulating *PRNP* expression, participates in lung cancer cell migration and invasion.

Considering miRNAs regulate numerous critical factors in tumorigenesis, the use of miRNAs in cancer therapy is attracting significant interest. In this study, we utilized a xenograft mouse model of lung cancer to demonstrate that consistent administration of the miR-193b-3p mimic led to a substantial inhibition of tumor growth, resulting in smaller tumor volumes when compared to the vector control (Fig. 3D). These findings indicate that miR-193b-3p may hold a therapeutic potential for lung cancer.

In the present study, analysis of tissue microarrays (TMAs) from stage I lung adenocarcinoma samples revealed a significant negative correlation between miR-193b-3p and PrPc expression, as determined by Spearman correlation analysis (*p* = 0.017) (Fig. 2E). Since all patients in our cohort were diagnosed with stage I lung adenocarcinoma, we were unable to assess how PrPc and miR-193b-3p expression varied across different stages of lung cancer. To address this limitation, we conducted a bioinformatics analysis using the TCGA dataset, which includes a more diverse population of lung adenocarcinoma patients at various stages. The results indicated that neither PrPc nor miR-193b-3p expression levels showed significant differences across the various stages of lung adenocarcinoma (data not shown), suggesting that these markers may not serve as reliable indicators of disease stage. Additionally, survival analysis in our cohort revealed that high PrPc expression or low miR-193b-3p expression was associated with poorer overall survival outcomes (Fig. 2F and H). These findings highlight that PrPc and miR-193b-3p may be involved in more complex biological processes that influence patient outcomes.

Although our study did not include experiments on normal lung tissues, several findings from previous research indicate that PrPc and miR-193b-3p exhibit distinct expression patterns between normal and cancerous lung tissues. The Human Protein Atlas reports that PrPc is expressed at low levels in normal lung tissue. Our previous work also found that PrPc is highly expressed in invasive adenocarcinomas compared to non-invasive and normal compartments [14]. Similarly, miR-193b expression is significantly downregulated in NSCLC tissues compared to adjacent normal tissues, as shown by quantitative RT-PCR analysis [34]. The differential expression patterns of PrPc and miR-193b-3p in cancerous versus non-cancerous lung tissues suggest that their regulatory mechanism is more prominent in lung cancer, supporting the critical role of miR-193b-3p in regulating PrPc expression in this context. In addition, disruption of this regulation, as shown by c-Jun overexpression in this study, led to downregulation of miR-193b-3p and an increase of PrPc expression, which may contribute to lung cancer progression. Further investigation of this regulatory mechanism in normal lung tissue and other relevant contexts could provide valuable insights into potential therapeutic strategies.

As miRNAs are essential for maintaining the precise regulation of cellular processes, their dysregulation can lead to abnormal cell growth and biosynthesis, ultimately contributing to tumor development, progression, and metastasis. The mechanisms associated with miRNAs dysregulation include abnormalities in transcriptional control of miRNA expression and miRNA biogenesis pathways as well as epigenetic methylation of miRNA loci [35]. Previous data have demonstrated that miR-193b was epigenetically silenced through promoter hyper-methylation in prostate cancer cell lines. This silencing resulted in the suppression of key prostate cancer subtype 1 (*PCS1*) genes, specifically *FOXM1* and *RRM2*, ultimately contributing to an aggressive phenotype [36, 37]. The mechanisms involved in the regulation of miR-193b expression, apart from promoter methylation, remain largely unknown.

In the present study, we did not find the promoter CpG islands of miR-193b-3p were hyper-methylated in both CL1-1 and CL1-5 cells (Supplementary Fig. S4), suggesting other mechanisms may be involved in regulating the differential expression of miR-193b-3p in CL1-1 and CL1-5 cells. Through promoter deletions reporter assays, bioinformatics analyses and differential gene expression profiling, we found that c-Jun may act as a transcriptional repressor of miR-193b-3p in lung cancer cells. Elevated c-Jun expression in CL1-1 cells led to suppression of miR-193b-3p expression while promoting PrPc expression (Fig. 4E and F, H. Conversely, knockdown of c-Jun in CL1-5 cells resulted in increased miR-193b-3p expression and a simultaneous downregulation of PrPc expression (Fig. 4G and H). We further demonstrated, using ChIP and luciferase assays with mutant constructs, that c-Jun directly binds to the miR-193b-3p promoter in lung cancer cells. The c-Jun binding site between positions −899 bp and −893 bp is crucial for the transcriptional repression of miR-193b-3p expression (Fig. 5B and C).

However, this finding appears to be at odds with the promoter deletion luciferase assay, which identified the −51 bp to + 437 bp region as the key regulatory sequence of the miR-193b-3p promoter (Fig. 4B). One possible explanation is that other transcriptional regulators or chromatin modifiers may cooperate with c-Jun to repress miR-193b-3p expression. In addition, we also observed that overexpression of c-Jun led to an increase in *PRNP* promoter activity (data not shown), suggesting that besides its negative regulation of miR-193b-3p expression, c-Jun may also play a direct role in controlling *PRNP* transcription.

In the present study, we demonstrate the functional link between c-Jun, miR-193b-3p, and PrPc in regulating lung cancer cell migration and invasion. In CL1-5 cells, silencing of *JUN* expression resulted in decreased cell migration and invasion, which was reversed by either PrPc overexpression or treatment with miR-193b-3p inhibitors (Fig. 5G and H). Together, these findings suggest that c-Jun, acting as a transcriptional repressor of miR-193b-3p, regulates *PRNP* expression and plays a key role in lung cancer metastasis. In addition to these in vitro insights, our tissue analysis of lung adenocarcinoma samples revealed a significant correlation between high expression levels of PrPc and/or c-Jun and poor overall survival in patients (Fig. 6B and D). This association suggests that the upregulation of PrPc and/or c-Jun may contribute to aggressive tumor behavior and worse clinical outcomes in lung cancer. Targeting the c-Jun/miR-193b-3p/PrPc axis could present a promising therapeutic avenue for inhibiting metastasis in lung adenocarcinoma.

The limitation of this study includes that our investigation focused solely on c-Jun-miR-193b-3p-PrPc axis in lung cancer, which may not fully capture the broader context in which other miRNAs and/or regulatory mechanisms could also be involved in modulating PrPc expression. Pease et al. by employing a sophisticated high-throughput arrayed screen, they found 13 miRNAs directly regulated *PRNP* expression by binding to 3’-UTR of its mRNA, leading to subsequent degradation. Additionally, four other miRNAs (miR-124-3p, miR-192-3p, miR-299-5p, and miR-376b-3p) were demonstrated to induce alterations in PrPc expression, potentially through interactions with indirect regulators [38]. Future research into other miRNAs and regulatory networks that influence PrPc in lung cancer could uncover valuable insights into cancer progression and identify potential therapeutic targets.

## Conclusions

**Fig. 7:**
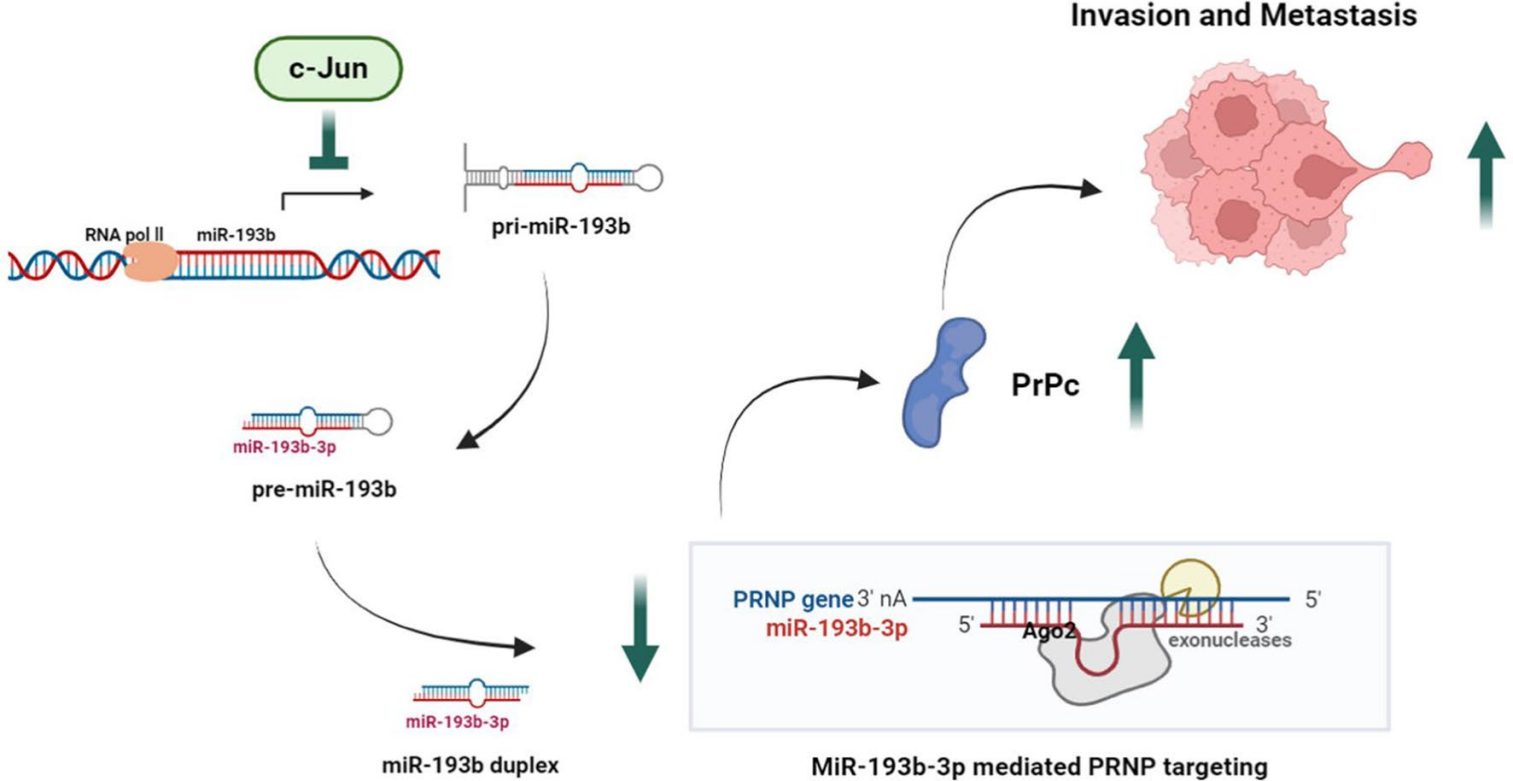
A schematic model illustrating the post-transcriptional regulation of *PRNP* expression in lung cancer. c-Jun functions as a transcriptional repressor of primary miR-193b (pri-miR-193b), resulting in the downregulation of miR-193b-3p expression. This downregulation subsequently leads to enhanced PrPc expression, potentially fostering increased invasion and metastasis of cancer cells. The observed molecular changes may contribute to a more aggressive cancer phenotype and worse patient survival, as demonstrated in the study

To the best of our knowledge, this study is the first to uncover a novel regulatory mechanism in which miR-193b-3p suppresses lung cancer metastasis by directly targeting the *PRNP* gene, and, for the first time, reveals that c-Jun transcriptionally represses miR-193b-3p, resulting in the upregulation of *PRNP* (Fig. 7). These findings provide crucial insights into the regulatory role of miRNAs in lung cancer migration and invasion, highlighting the potential of targeting the c-Jun/miR-193b-3p/*PRNP* axis as a promising strategy for inhibiting lung cancer progression and metastasis.

## Supplementary Information

Additional file 1.

Additional file 2.

## Data Availability

The datasets used and/or analyzed during the current study are available from the corresponding author on reasonable request.

## Abbreviations

RT-qPCR: Reverse transcription quantitative polymerase chain reaction
MiRNAs: MicroRNAs
Pri-miR-193b: Primary miR-193b
Pre-miR-193b: Precursor miR-193b
NSCLC: Non-small cell lung cancer
OS: Overall survival
DFS: Disease-free survival
TMA: Tissue microarray
VGH-Taipei: Taipei Veterans General Hospital
RIP: RNA immunoprecipitation
ChIP: Chromatin immunoprecipitation
PBS: Phosphate-buffered saline
LNA-ISH: LNA-in situ hybridization
IHC: Immunohistochemistry
WT: Wild-type
Mut: Mutant
uPA: Urokinase type plasminogen activator
DDAH1: Dimethylaminohydrolase 1
CCND1: Cyclin D1
PAX5: Paired box 5
RELA: V-rel reticuloendotheliosis viral oncogene homolog A
GR: Glucocorticoid receptor
