# Supplementary material for: miR-193b-3p suppresses lung cancer cell migration and invasion through PRNP targeting
Source: J Biomed Sci. 2025 Feb 20;32:28. doi: 10.1186/s12929-025-01121-1 (PMC11841292; doi:10.1186/s12929-025-01121-1)
Supplement: Supplementary file 1 — Additional file 1. [file 12929_2025_1121_MOESM1_ESM.pptx]

## Slide 1
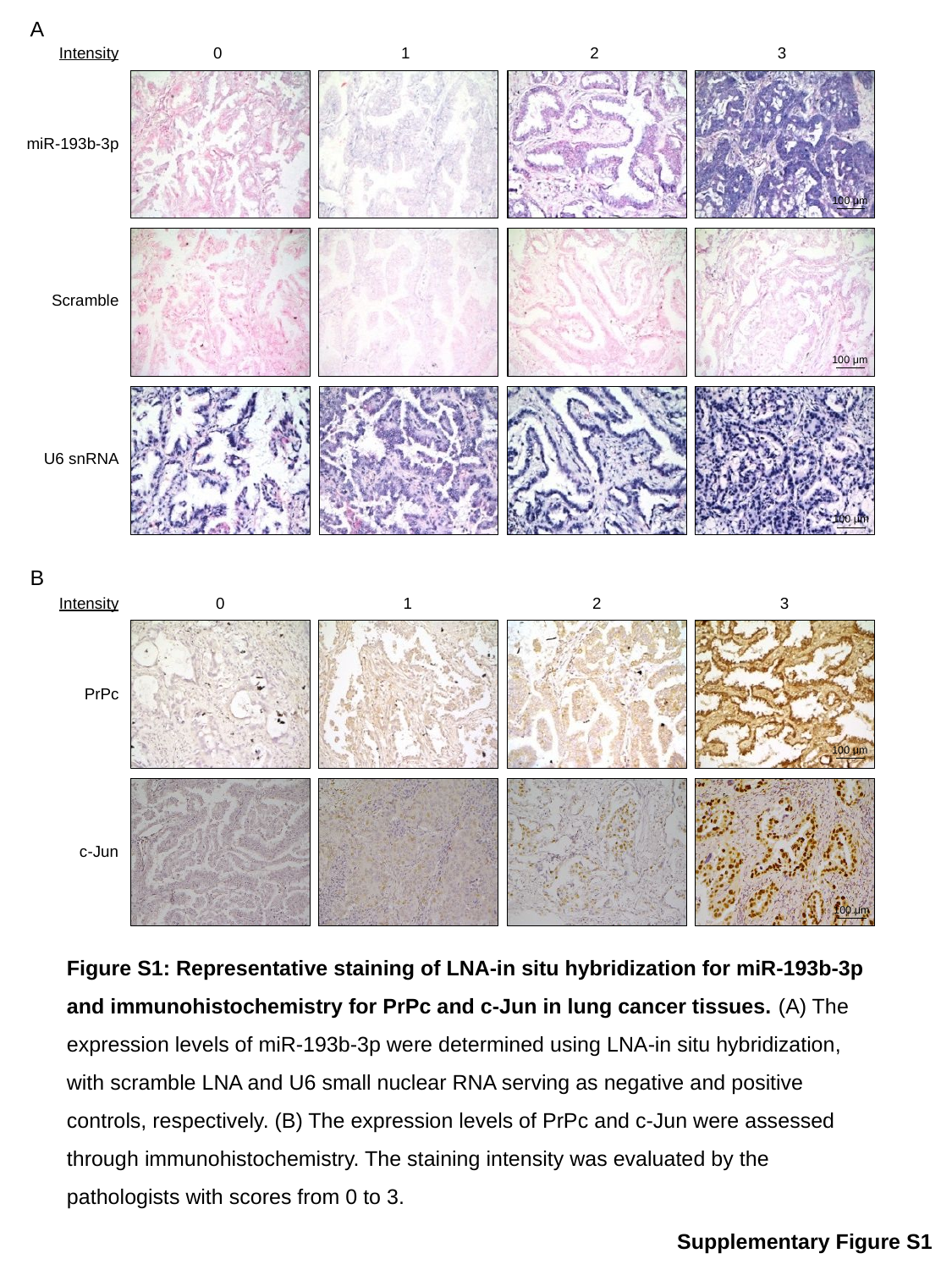

A
Intensity
0
1
2
3
100 μm
miR-193b-3p
Scramble
100 μm
U6 snRNA
100 μm
B
Intensity
0
1
2
3
PrPc
100 μm
c-Jun
100 μm
Figure S1: Representative staining of LNA-in situ hybridization for miR-193b-3p and immunohistochemistry for PrPc and c-Jun in lung cancer tissues. (A) The expression levels of miR-193b-3p were determined using LNA-in situ hybridization, with scramble LNA and U6 small nuclear RNA serving as negative and positive controls, respectively. (B) The expression levels of PrPc and c-Jun were assessed through immunohistochemistry. The staining intensity was evaluated by the pathologists with scores from 0 to 3.
Supplementary Figure S1

## Slide 2
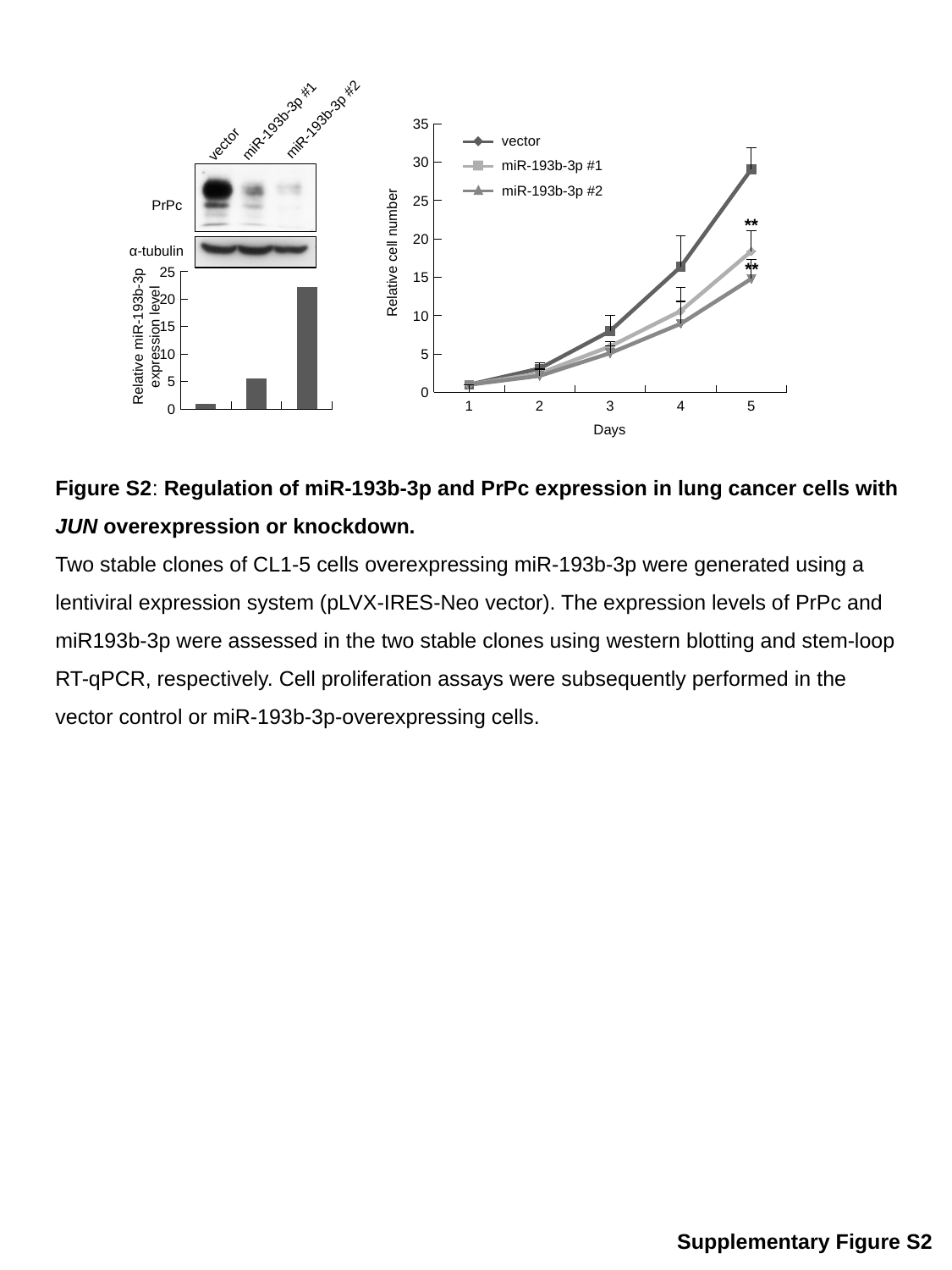

miR-193b-3p #2
### Chart
| Category | vector | miR-193b #1 | miR-193b #2 |
|---|---|---|---|miR-193b-3p #1
vector
miR-193b-3p #1
miR-193b-3p #2
vector
PrPc
**
α-tubulin
Relative cell number
**
### Chart
| Category | |
|---|---|Relative miR-193b-3p
expression level
Days
Figure S2: Regulation of miR-193b-3p and PrPc expression in lung cancer cells with JUN overexpression or knockdown.
Two stable clones of CL1-5 cells overexpressing miR-193b-3p were generated using a lentiviral expression system (pLVX-IRES-Neo vector). The expression levels of PrPc and miR193b-3p were assessed in the two stable clones using western blotting and stem-loop RT-qPCR, respectively. Cell proliferation assays were subsequently performed in the vector control or miR-193b-3p-overexpressing cells.
Supplementary Figure S2

## Slide 3
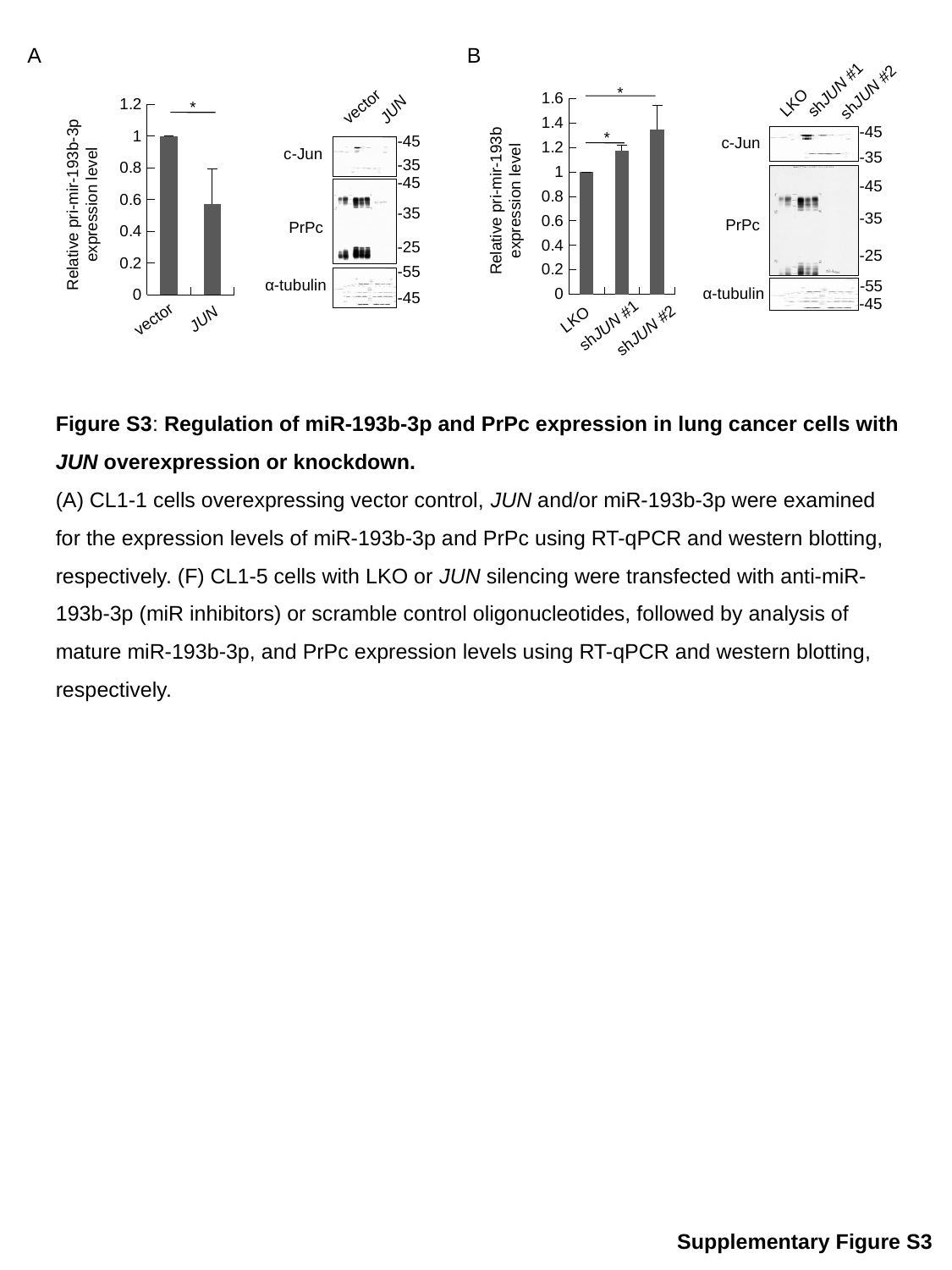

A
B
shJUN #1
shJUN #2
LKO
-45
-35
-45
-35
-25
-55
-45
c-Jun
PrPc
α-tubulin
### Chart
| Category | |
|---|---|*
*
Relative pri-mir-193b expression level
LKO
shJUN #1
shJUN #2
vector
JUN
-45
-35
-45
-35
-25
-55
-45
c-Jun
PrPc
α-tubulin
### Chart
| Category | |
|---|---|*
Relative pri-mir-193b-3p
expression level
vector
JUN
Figure S3: Regulation of miR-193b-3p and PrPc expression in lung cancer cells with JUN overexpression or knockdown.
(A) CL1-1 cells overexpressing vector control, JUN and/or miR-193b-3p were examined for the expression levels of miR-193b-3p and PrPc using RT-qPCR and western blotting, respectively. (F) CL1-5 cells with LKO or JUN silencing were transfected with anti-miR-193b-3p (miR inhibitors) or scramble control oligonucleotides, followed by analysis of mature miR-193b-3p, and PrPc expression levels using RT-qPCR and western blotting, respectively.
Supplementary Figure S3

## Slide 4
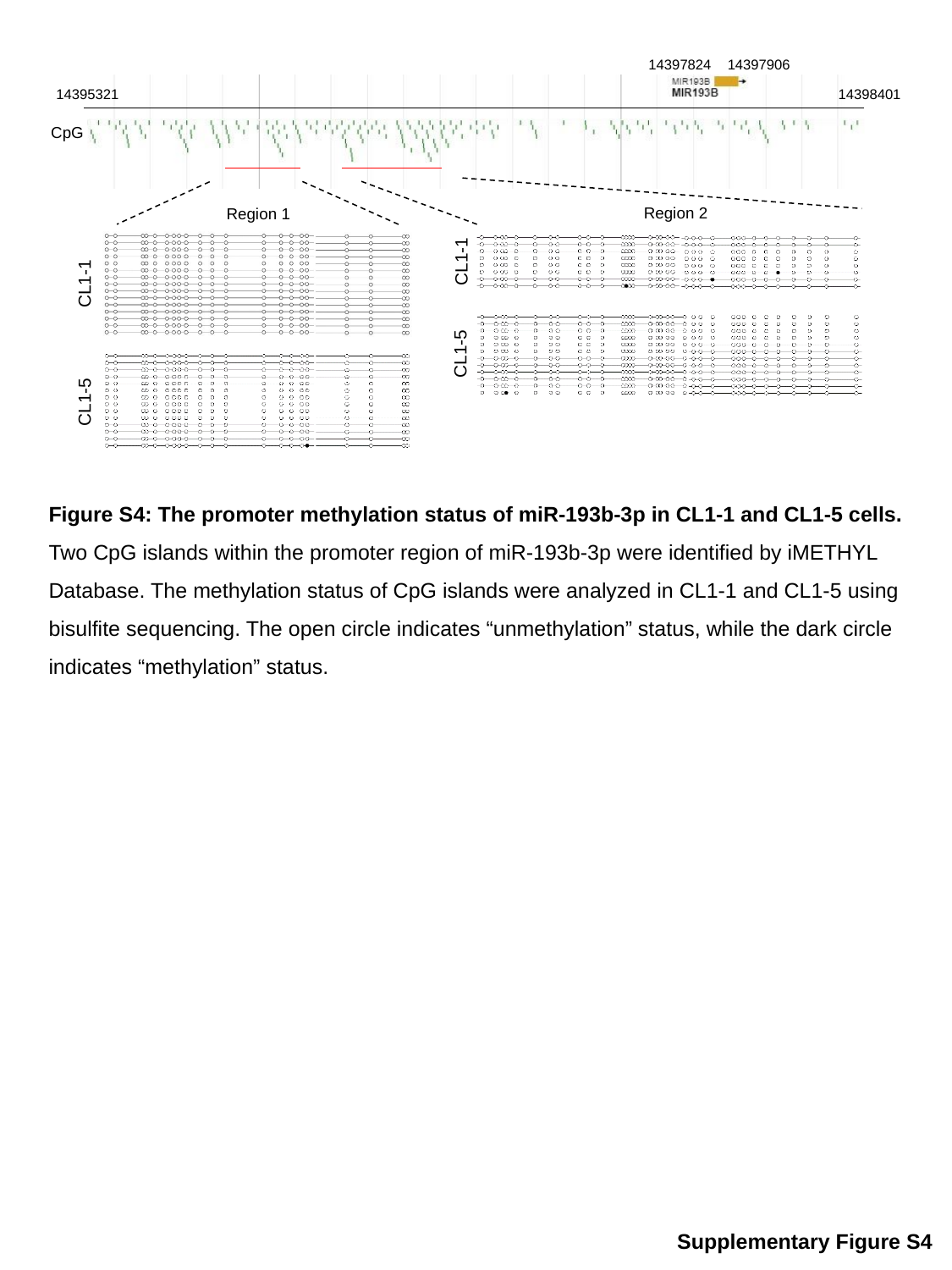

14397906
14397824
14395321
14398401
CpG
Region 2
Region 1
CL1-1
CL1-1
CL1-5
CL1-5
Figure S4: The promoter methylation status of miR-193b-3p in CL1-1 and CL1-5 cells. Two CpG islands within the promoter region of miR-193b-3p were identified by iMETHYL Database. The methylation status of CpG islands were analyzed in CL1-1 and CL1-5 using bisulfite sequencing. The open circle indicates “unmethylation” status, while the dark circle indicates “methylation” status.
Supplementary Figure S4
