# Supplementary material for: miR-193b-3p suppresses lung cancer cell migration and invasion through PRNP targeting
Source: J Biomed Sci. 2025 Feb 20;32:28. doi: 10.1186/s12929-025-01121-1 (PMC11841292; doi:10.1186/s12929-025-01121-1)
Supplement: Supplementary file 2 — Additional file 2. [file 12929_2025_1121_MOESM2_ESM.docx]

**Supplementary materials**

**Genomic Bisulfite Sequencing for the promoter region of miR-193b-3p**

Genomic DNA from CL1-1 and CL1-5 was extracted by HiYield^TM^ Genomic DNA Extraction Kit (Arrowtec Life Science, cat# YGT50), and samples were subjected to CT conversion using EZ DNA Methylation-Gold™ Kit (Zymo Research, cat# D5005) according to the manufacturer’s instructions. Bisulfite-treated genomic DNA was subjected to PCR with the following primer sets: set 1, CpG193b1F: 5’-GGAGGATTTAAGGTTGGGTTTTAAT-3’ and CpG193b1R: 5’-CCTAAAAACTTAAACACCCACCTAC-3’; set 2, CpG193b2F: 5'-TAGTGTTTGGGTTTGGAAATTGA-3' and CpG193b2R: 5'-AAAAAAAACTAACAACTTTAAAAAAC-3'. PCR fragments were cloned into the yT&A cloning vector (Violet Bioscience Inc.), and independent clones were sequenced.

**Table S1. Primers used for deletion constructs of 3’-UTR of *PRNP***

| Primers | Sequence (5' to 3') |
| --- | --- |

| PUTR-F1 | GGGACTAGTGGAAGGTCTTCCTGTTTTCAC |
| --- | --- |
| PUTR-F2 | GGGACTAGTGGCACTGGAAAACATAGAG |
| PUTR-F3 | GGGACTAGTTAGTGCAACAGGTTGAGGCT |
| PUTR-R1 | GGGACTAGTGGTGCTCATCTTCGCTCGTT |
| PUTR-R2 | GGGACTAGTTATTTCTGTCATCTCCAACCT |
| PUTR-R3 | GGGACTAGTTGCACACTGACCATT |
| PUTR-R4 | GGGACTAGTTCAGCTGCCTTAATTACC |
| PUTR-R5 | GGGACTAGTCTGTGAATATGTCCTCTAGC |
| PUTR-R6 | GGGACTAGTTCCCAAGAGCTAAGA |

Forward (F) and reverse (R) primers were designed with SpeI restriction enzyme site for ligation of PCR fragments into pGL3-control vector at XbaI site. Primer pairs used to amplify different fragments of 3’-UTR of *PRNP* were described as follow: PUTR-F1 and PUTR-R1 for the 1-1605 fragment, PUTR-F1 and PUTR-R2 for the 1-1126 fragment, PUTR-F1 and PUTR-R3 for the 1-976 fragment, PUTR-F1 and PUTR-R4 for the 1-829 fragment, PUTR-F1 and PUTR-R5 for the 1-676 fragment, PUTR-F1 and PUTR-R6 for the 1-526 fragment, PUTR-F2 and PUTR-R4 for the 147-829 fragment, and PUTR-F3 and PUTR-R4 for the 287-829 fragment.

**Table S2. Primers used for construction of plasmids expressing miR-193b-3p and putative transcription factors**

| Primers | Sequence (5' to 3') |
| --- | --- |
| miR-193b-F1 and R1^a^ | (Forward) GGGAATTCCAGCCATGGTGTGGCAAATGTC |
|  | (Reverse) GGGTACCTTCGGCAAACTGAGAACACA |
| ETS1^b^ | (Forward) GCAAGCTTATGAGCTACTTTGTGGATTCTGCTGGG |
|  | (Reverse) GCGCGGCCGCTCACTCGTCGGCATCTGGCTTGACGT |
| GR^c^ | (Forward) CGGTACCAATGGACTCCAAAGAATCATTA |
|  | (Reverse) CTCTAGATCACTTTTGATGAAACAGAAG |
| c-Jun^d^ | (Forward) GCAAGCTTATGACTGCAAAGATGGAAACGA |
|  | (Reverse) GCGAATTCTCAAAATGTTTGCAACTGCTGC |

^a^ The miR-193b forward (F1) and reverse (R1) primers were designed with EcoRI and KpnI restriction enzyme sites, respectively, for ligation of PCR fragments into pCMV-HA vector. EcoRI and BamHI restriction enzyme sites were used from yT&A vector to ligate to pLVX-IRES-Neo vector for subsequent lentivirus production.

^b^ The ETS1 forward (F) and reverse (R) primers were designed with HindIII and NotI restriction enzyme sites, and subsequently the PCR fragments were constructed into pcDNA3.1 myc-His C vector; ^c^ The GR forward (F) and reverse (R) primers were designed with KpnI and XbaI restriction enzyme sites, and subsequently the PCR fragments were constructed into pFLAG-CMV-2 vector.

^d^ The c-Jun forward (F) and reverse (R) primers were designed with HindIII and EcoRI restriction enzyme sites, and subsequently the PCR fragments were constructed into pcDNA3.1 myc-His C vector.

**Table S3. Primers used for deletion constructs of the miR-193b-3p promoter**

| Primers | Sequences (5' to 3') |
| --- | --- |

| PmiRP-F1 | GGGTACCCCTGGGACCTCCTCTGTGGCATCAGGAC |
| --- | --- |
| PmiRP-F2 | GGGTACCGGCATTGGCACTGGGTGTTCC |
| PmiRP-F3 | GGGTACCTTGTGGCGGCAGCGGACCGTGGA |
| PmiRP-R1 | GCTCGAGCCAGACAAGCGTCTCGCGCCGAGTCC |
| PmiRP-R2 | GCTCGAGTCCACGGTCCGCTGCCGCCACAA |

Forward (F) and reverse (R) primers were designed with KpnI and XhoI restriction enzyme site, respectively, for ligation of PCR fragments into pGL3-basic vector. The amplification of various deletion constructs of miR-193b-3p promoter was carried out using specific primer pairs: PmiRP-F1 and PmiRP-R1 for the -1362/+437 fragment, PmiRP-F2 and PmiRP-R1 for the -607/+437 fragment, PmiRP-F3 and PmiRP-R1 for the -51/+437 fragment, and PmiRP-F1 and PmiRP-R2 for the -1362/-24 fragment.

**Table S4. Primers used for real-time PCR**

| Primers | Sequences (5' to 3') |
| --- | --- |

| E2F1-F | ATGTTTTCCTGTGCCCTGAG |
| --- | --- |
| E2F1-R | ATCTGTGGTGAGGGATGAGG |
| PAX5-F | TTGGAGGATCCAAACCAAAG |
| PAX5-R | CACGGTGTCATTGTCACACA |
| RELA-F | TCTGCTTCCAGGTGACAGTG |
| RELA-R | ATCTTGAGCTCGGCAGTGTT |
| ETS1-F | TGACTACCCCTCGGTCATTC |
| ETS1-R | GTTGTCTGGGGTGACGACTT |
| GR-F | GAGAGGGGAGATGTGATGGA |
| GR-R | ATTGAGAAGCGACAGCCAGT |
| TP53-F | GTTCCGAGAGCTGAATGAGG |
| TP53-R | TCTGAGTCAGGCCCTTCTGT |
| C-JUN-F | CCCCAAGATCCTGAAACAGA |
| C-JUN-R | CCGTTGCTGGACTGGATTAT |
| PRNP-F | AATCAAGCAGCACACGGTCA |
| PRNP-R | TCGGTGAAGTTCTCCCCCTT |
| GAPDH-F | TGGGCTACACTGAGCACCAG |
| GAPDH-R | CAGCGTCAAAGGTGGAGGAG |

**Table S5. Primers for chromatin immunoprecipitation (ChIP) analysis**

| Primers | Sequences (5' to 3') |
| --- | --- |

| miR193-CHIP F1 | CGTGACCTGTGTTGGCCAACAG |
| --- | --- |
| miR193-CHIP R1 | GACATGACTTGTCCCTCTCCAC |
| miR193-CHIP F2 | GTTCCAGCACCTCTCCAAGAGC |
| miR193-CHIP R2 | CACAGACACACTGTGTTCCCAAG |
| miR193-CHIP F3 | CTGCGGTGGTACTGGAAGTG |
| miR193-CHIP R3 | CGCCTTCCTAGAAACGCTCG |
| miR193-CHIP F4 | CTCCAATCGAGGCGGAGGCTG |
| miR193-CHIP R4 | CACCCACCTGCCGCGATCCCT |

**Table S6. Putative *PRNP*-targeting miRNAs**

| **miRNA** | **No. of binding sites** | **Prediction tool** |
| --- | --- | --- |
| miR-216b-5p | 2 | W, M, R |
| miR-22-3p | 1 | M, R |
| miR-193a-3p | 1 | M, R |
| miR-193b-3p | 1 | M, R |
| miR-653-5p | 1 | W, R |
| miR-1290-3p | 1 | W, M |

Abbreviations:

W, miRWalk; M, miRDB; R, miRanda
